# Substantial and sustained improvement of serrated polyp detection after a simple educational intervention: results from a prospective controlled trial

**DOI:** 10.1136/gutjnl-2019-319804

**Published:** 2020-03-05

**Authors:** Arne G C Bleijenberg, Monique E van Leerdam, Marloes Bargeman, Jan Jacob Koornstra, Yasmijn J van Herwaarden, Manon CW Spaander, Silvia Sanduleanu, Barbara A J Bastiaansen, Erik J Schoon, Niels van Lelyveld, Evelien Dekker, Joep E G IJspeert

**Affiliations:** 1 Department of Gastroenterology and Hepatology, Amsterdam UMC, University of Amsterdam, Amsterdam, The Netherlands; 2 Department of Gastroenterology and Hepatology, Netherlands Cancer Institute, Amsterdam, The Netherlands; 3 Department of Gastroenterology and Hepatology, Medical Spectrum Twente, Enschede, The Netherlands; 4 Department of Gastroenterology and Hepatology, University Medical Center Groningen, Groningen, The Netherlands; 5 Department of Gastroenterology and Hepatology, Radboud University Medical Center, Nijmegen, The Netherlands; 6 Department of Gastroenterology and Hepatology, Erasmus University Medical Center, Rotterdam, The Netherlands; 7 Department of Gastroenterology and Hepatology, Maastricht University Medical Center, Maastricht, The Netherlands; 8 Department of Gastroenterology and Hepatology, Catharina Hospital, Eindhoven, The Netherlands; 9 Department of Gastroenterology and Hepatology, Saint Antonius Hospital, Nieuwegein, The Netherlands

**Keywords:** colorectal cancer, colonoscopy, colonic polyps

## Abstract

**Objective:**

Serrated polyps (SPs) are an important cause of postcolonoscopy colorectal cancers (PCCRCs), which is likely the result of suboptimal SP detection during colonoscopy. We assessed the long-term effect of a simple educational intervention focusing on optimising SP detection.

**Design:**

An educational intervention, consisting of two 45 min training sessions (held 3 years apart) on serrated polyp detection, was given to endoscopists from 9 Dutch hospitals. Hundred randomly selected and untrained endoscopists from other hospitals were selected as control group. Our primary outcome measure was the proximal SP detection rate (PSPDR) in trained versus untrained endoscopists who participated in our faecal immunochemical test (FIT)-based population screening programme.

**Results:**

Seventeen trained and 100 untrained endoscopists were included, who performed 11 305 and 51 039 colonoscopies, respectively. At baseline, PSPDR was equal between the groups (9.3% vs 9.3%). After training, the PSPDR of trained endoscopists gradually increased to 15.6% in 2018. This was significantly higher than the PSPDR of untrained endoscopists, which remained stable around 10% (p=0.018). All below-average (ie, PSPDR ≤6%) endoscopists at baseline improved their PSPDR after training session 1, as did 57% of endoscopists with average PSPDR (6%–12%) at baseline. The second training session further improved the PSPDR in 44% of endoscopists with average PSPDR after the first training.

**Conclusion:**

A simple educational intervention was associated with substantial long-term improvement of PSPDR in a prospective controlled trial within FIT-based population screening. Widespread implementation of such interventions might be an easy way to improve SP detection, which may ultimately result in fewer PCCRCs.

**Trial registration number:**

NCT03902899.

**Significance of this study**

**What is already known on this subject?**

- Serrated polyps are precursor lesions to colorectal cancer, but are detected suboptimally by endoscopists, which might lead to postcolonoscopy colorectal cancers.

**What are the new findings?**

- Implementation of a brief and relatively simple educational intervention in a population screening setting resulted in substantial and long-term improvement of the detection of serrated polyps.

**How might it impact on clinical practice in the foreseeable future?**

- Adoption of this brief educational intervention in training and accreditation programmes could be a simple method to improve quality and effectivity of colonoscopy.

## Introduction

Colorectal cancer (CRC) is a leading causes of cancer-related mortality worldwide,^1 2^ and arises from premalignant polyps.^3^ CRC incidence can be reduced dramatically by removal of these polyps.^4^ Adenomatous polyps have long been considered the only precursor lesion for CRC, and therefore CRC prevention has traditionally focused on detection and removal of adenomas. However, recent evidence shows that up to one-third of CRCs arise through serrated polyps (SP).^5–7^ This has caused a paradigm shift in CRC prevention; endoscopists should nowadays also detect and resect these SPs.^8 9^

However, due to the relative recent discovery of their malignant potential, the clinical relevance and appearance of SPs might be less well-known among endoscopists. In addition, detection of SPs is more difficult due to their flat shape, pale colour and vague borders. Furthermore, mucus is often attached to the polyp, thereby hiding it from the endoscopist’s eye.^9 10^ Two studies reported proximal SP detection rate (PSPDR) ranging from 1%–2% in low-detecting endoscopists up to 18% in high-detecting endoscopists,^11 12^ suggesting that low detectors frequently miss SPs.

Poor detection therefore seems to be one of the main challenges in the current management of SPs. This is illustrated by the disproportionally high contribution of SPs to the development of postcolonoscopy CRC (PCCRC): both molecular characteristics as well as the preferential right-sided location of PCCRCs suggest that PCCRCs disproportionally often arise from missed or incompletely resected SPs.^13–16^

Analogous to the risk reduction of PCCRCs at increased adenoma detection rate (ADR),^17 18^ it therefore seems possible that an increase in SP detection will result in a decline in PCCRCs. Educational interventions aiming to improve adenoma detection have resulted in an increased ADR,^19–21^ but not in increased SP detection rates.^22^ Hence, dedicated interventions specifically aiming to improve SP detection seem warranted. To our knowledge, the effect of such educational interventions has not been studied.

We performed a study to evaluate whether a simple and easily applicable educational intervention consisting of two 45 min training sessions (approximately 3 years apart) on optimising SP detection, could lead to a long-term increase in SP detection in a large prospective controlled trial nested within the Dutch faecal immunochemical test (FIT)-based CRC screening programme.

## Methods

### Study design

In this prospective trial, we assessed the effect of a simple educational intervention on the detection of SPs in the Dutch nationwide CRC screening programme. All residents of the Netherlands aged 55–75 years are invited 2-yearly to perform an FIT. All individuals with a positive test result are invited for follow-up by colonoscopy. Each year, all residents from the same birth year are invited, thereby assuring comparable invitees throughout the country.

All endoscopists from nine Dutch hospitals spread throughout the Netherlands, performing colonoscopies in FIT-positives in the Dutch national screening programme, were invited to receive our educational intervention and were thus allocated to the intervention arm. This intervention consisted of two 45 min training sessions; one in 2014 and another one 3 years later (for details see paragraph ‘ Educational Intervention’ below). The selection of these nine centres was pragmatic, and mainly based on scattered location throughout the Netherlands. Hundred randomly selected endoscopists employed in other hospitals throughout the Netherlands were selected as controls.

Our study fell beyond the Dutch legislation regarding Medical Research Involving Human Subjects Act since patients were not exposed to any intervention, and because their privacy was guaranteed by anonymisation of the database.^23 24^ Patients were not involved in the design and conduct of this study.

### Inclusion criteria

Endoscopists were eligible for inclusion if they were accredited to perform colonoscopies within the Dutch FIT-based screening programme. This meant all included endoscopists were subjected to strict quality monitoring and auditing throughout the study duration, which have been described in detail previously.^25^ Briefly, among several other quality criteria, each endoscopist had to perform ≥200 colonoscopies per year, ≥50 polypectomies per year and achieve a caecum intubation rate of ≥95%, withdrawal time of ≥6 min in ≥90% of colonoscopies, adenoma detection rate of ≥30%, removal of ≥90% of detected polyps and retrieval for pathological examination of ≥90% of resected polyps in screening colonoscopies.^25^ All screening colonoscopies performed by the included endoscopists were analysed to calculate our primary and secondary end points.

### Exclusion criteria

Endoscopists allocated to the intervention arm who did not attend both training sessions were excluded for primary analysis. We performed a sensitivity analysis in which endoscopists that attended only one training session were included in the intervention group. Endoscopists who did not perform screening colonoscopies before the first training session were excluded, since no baseline detection rates of these endoscopists could be established.

### Educational intervention

The educational intervention we implemented consisted of two training sessions. All endoscopists in the intervention arm received these two training sessions; one in 2014 and a second session in 2017 (two hospitals received the latter in October and November 2016, respectively). Training sessions were given by one of three research fellows specialised in serrated polyps (JEGI, AGCB and YJvH).

Training session 1 and 2 were almost identical. Both consisted of an oral presentation of about 45 min. The training sessions primarily focused on making endoscopists aware of the importance of diagnosing, detecting and resecting SPs. Statistics about the contribution of SPs to sporadic and postcolonoscopy CRC were presented, and typical features of the different SP subtypes (hyperplastic polyps (HPs), sessile serrated lesions (SSLs) and traditional serrated adenomas (TSAs)) were highlighted. Optical diagnosis of colorectal polyps using the Workgroup serrAted polypS and Polyposis classification was discussed.^26^ The slides of these presentations are available in online supplementary material.

10.1136/gutjnl-2019-319804.supp1 Supplementary data

### Data collection: Dutch CRC screening programme

Data from the national CRC screening programme were used for our analyses. All colonoscopies in FIT-positives are routinely registered in this centralised database, which is coordinated by the governmental National Institute for Public Health and the Environment (Rijksinstituut voor Volksgezondheid en Milieu (RIVM)). This database includes detailed information of every colonoscopy performed in the population screening programme in the Netherlands, such as the endoscopist performing the endoscopy, caecal intubation, withdrawal time, polyp detection and resection. Histological data were extracted from PALGA, the nationwide network and registry of histopathology and cytopathology in The Netherlands.^27^

For the purpose of this study, we extracted from this database all colonoscopies that were performed between January 2014 and September 2018 by the included endoscopists.

All identifying variables were removed by employees of the RIVM before the data were made available to us in order to comply with privacy legislation of the European General Data Protection Regulation Act.^24^ As such, no personal information about endoscopists (eg, age, gender, years of experience) could be included in the provided data.

### Outcome parameters

The primary outcome measure was the difference in PSPDR in colonoscopies performed by trained versus untrained endoscopists based on all colonoscopies performed by trained endoscopists after they received their first training, and all colonoscopies performed by the untrained endoscopists in the control arm. PSPDR was defined as the percentage of patients in whom at least one SP proximal to the descending colon was removed.^12^ The difference was expressed as absolute percentage and as OR for the detection of a proximal SP by control group versus the intervention group.

Secondary outcome measures included the detection rates of subtypes of SPs (ie, HP, SSL, and TSA) and (advanced) adenomas.

Another secondary outcome measure was the effect of our intervention on individual endoscopists. For this purpose, baseline detection rates were measured using all colonoscopies prior to the first training session in 2014. The endoscopists were divided into three groups based on their baseline PSPDR: below-average (PSPDR ≤6%), average (PSPDR 6%–12%) and above-average (PSPDR ≥12%). The cut-off PSPDR values for these three groups were chosen arbitrarily based on baseline PSPDR. Baseline detection rates were compared with those after the first and the second training, respectively, for each individual endoscopist. The effect was assessed by quantifying how many endoscopists improved, remained stable or declined in PSPDR over time, with the three baseline strata as benchmark (ie, below-average, average and above-average).

### Statistical analyses

Detection rates of trained and untrained endoscopists were compared using mixed-effects logistic regression analysis. Training was analysed as a fixed effect into the model. To adjust for the fact that our data were clustered on endoscopist-level and for the fact that colonoscopies were performed in different types of centres (ie, academic hospital, general hospital, private clinic), we added random intercepts in the model for the endoscopist and the type of centre.

Differences in colonoscopy and patient characteristics were assessed using Mann-Whitney U tests (non-parametric numerical variables) and χ^2^ tests (discrete variables). All analyses were performed using Statistical Package for Social Sciences V.24 (IBM, Somers, New York, USA) and RStudio^28^ with *lme4* package,^29^ with the function of *glmer*. Figures were produced using GraphPad Prism (V.7.03, GraphPad Software, La Jolla, California, USA).

Since at the time of initiation of our study in 2014 too little evidence was available about baseline PSPDR in a FIT-based cohort, and because we were unable to make a reliable estimation about the number of colonoscopies per endoscopist, it was difficult to reliably produce the required parameters for sample size calculations. Instead, we therefore aimed to include as many endoscopists as possible in the intervention arm by selecting a relatively large number of hospitals for our intervention. The large number of control endoscopists was chosen pragmatically.

## Results

### Baseline characteristics

A total of 34 endoscopists from 9 centres were potentially eligible for inclusion in the intervention arm. Eleven were excluded due to absence during one of the training sessions, while six were excluded because they did not yet perform colonoscopies for the population screening programme at the start of the study and thus no baseline PSPDR could be measured. Thus, 17 endoscopists attended both training sessions and were included in the intervention arm. In addition, 100 randomly selected untrained endoscopists were included as controls (table 1). Three trained endoscopists (18%) were employed in an academic hospital, 10 (59%) in a general hospital and 4 (23.5%) worked mainly in a private clinic. In the control group, 3 (3%) of the untrained endoscopists worked in an academic centre, while 91 (91%) and 6 (6%) were employed by general and private clinics, respectively (table 1).

**Table 1:** Characteristics of endoscopists and colonoscopies

Included endoscopists (n=117)			
	Overall (n=117)	Intervention arm (n=17)	Control arm (n=100)
Type of centre of endoscopist, n (%)			
Academic hospital	6 (5.1%)	3 (18%)	3 (3.0%)
General hospital	101 (86%)	10 (59%)	91 (91%)
Private clinic	10 (8.5%)	4 (23.5%)	6 (6.0%)

Pretraining colonoscopies* (n=6997) Characteristics of scoped patients				
Overall (n=6997)	Intervention arm (n=928)	Control arm (n=6069)	P value
Age, mean (SD)	71 (SD 5.1)	72 (SD 4.7)	71 (SD 5.1)	<0.001
Male gender, n (%)	4119 (59%)	563 (61%)	3556 (59%)	0.24
Type of centre, n (%)				<0.001
Academic hospital	940 (13%)	226 (24%)	714 (12%)	
General hospital	5357 (77%)	574 (62%)	4783 (79%)	
Private clinic	700 (10%)	128 (14%)	572 (9.4%)	
Quality parameters, n (%)				
Caecal intubation, n (%)	6642 (95%)	882 (95%)	5760 (95%)	1
Inspection time, median (IQR)	14 (9–21)	16 (10–26)	13 (9–20)	<0.001
Inspection time ≥6 min	6541 (94%)	912 (98%)	5629 (93%)	<0.001
BBPS ≥2 in all segments	6727 (96%)	896 (96%)	5831 (96%)	0.94

Post-training colonoscopies* (n=55 344) Characteristics of scoped patients				
	Overall (n=55 344)	Intervention arm (n=10 377)	Control arm (n=44 967)	P value
Age, mean (SD)	66 (SD 5.3)	66 (SD 5.1)	66 (SD 5.4)	0.005
Male gender, n (%)	32 587 (59%)	6053 (58%)	26 534 (59%)	0.21
Type of centre, n (%)				<0.001
Academic hospital	3516 (6.4%)	2057 (20%)	1459 (3.2%)	
General hospital	42 705 (79%)	5993 (58%)	37 712 (84%)	
Private clinic	8123 (15%)	2327 (22%)	5796 (13%)	
Year of colonoscopy, n (%)				
2014	616 (1.1%)	616 (5.9%)*	Not applicable*	
2015	13 107 (24%)	2687 (26%)	10 420 (23%)	
2016	15 216 (27.5%)	2921 (28%)	12 295 (27%)	
2017	16 437 (30%)	2700 (26%)	13 737 (30.5%)	
2018	9968 (18%)	1453 (14%)	8515 (19%)	<0.001
Quality parameters, n (%)				
Caecal intubation, n (%)	53 844 (97%)	10 061 (97%)	43 783 (97%)	0.02
Inspection time, median (IQR)	14 (10–21)	17 (12–26)	13 (9–20)	<0.001
Inspection time ≥6 min	53 973 (97.5%)	10 276 (99%)	43 697 (97%)	<0.001
BBPS ≥2 in all segments	52 896 (96%)	9818 (95%)	43 078 (96%)	<0.001

Percentages ≥10% are rounded to the nearest integer.

*Pretraining refers to baseline colonoscopies performed in 2014 prior to the first training session (trained endoscopists), or all colonoscopies in 2014 (untrained endoscopists). Post-training refers to all colonoscopies after the first training (trained endoscopists), or all colonoscopies from 2015 onwards (untrained endoscopists).

BBPS, Boston Bowel Preparation Score.

Endoscopists in the intervention arm performed a total of 928 baseline colonoscopies (ie, colonoscopies in 2014 prior to the first training session), while endoscopists in the control arm performed 6069 baseline colonoscopies (ie, colonoscopies in 2014; table 1). Age and gender distribution of patients were similar between the intervention and control arm, but median inspection time of colonoscopy was longer (16 vs 13 min, p<0.001) for colonoscopies performed by endoscopists in the intervention arm. Detection rates at baseline did not differ across all different polyp subtypes (table 2).

**Table 2:** Baseline detection rates based on 928 colonoscopies by trained endoscopists and 6069 colonoscopies by untrained endoscopists*

	Endoscopists in intervention arm (n=17)	Endoscopists in control arm (n=100)	P value†
≥1 Proximal SP (=PSPDR), % (95% CI)	9.3% (7.4% to 11%)	9.3% (8.6% to 10%)	0.48
≥1 SP	23% (20% to 26%)	24% (23% to 25%)	0.87
≥1 HP	20% (17% to 22%)	20% (19% to 21%)	0.61
≥1 SSL	5.9% (4.4% to 7.4%)	5.4% (4.9% to 6.0%)	0.72
≥1 TSA	1.1% (0.4% to 1.7%)	1.4% (1.1% to 1.8%)	0.49
≥1 Adenoma (=ADR), % (95% CI)	66% (63% to 70%)	66% (65% to 68%)	0.99
≥1 Advanced adenoma	41% (38% to 44%)	45% (44% to 47%)	0.13

Percentages ≥10% are rounded to the nearest integer.

*Based on colonoscopies in 2014 prior to the first training session (trained endoscopists), or *all* colonoscopies in 2014 (untrained endoscopists).

†P values are based on mixed-effects logistic regression analyses, with fixed effects for training and adjusted with random intercepts for type of centre and endoscopist.

ADR, adenoma detection rate; HP, hyperplastic polyp; PSPDR, proximal serrated polyp detection rate; SP, serrated polyp; SSL, sessile serrated lesion; TSA, traditional serrated adenoma.

After these baseline colonoscopies, trained and untrained endoscopists performed 10 377 and 44 967 post-training colonoscopies, respectively (table 1). Age and gender distribution of patients were similar, but colonoscopies performed by trained endoscopists had a longer median withdrawal time (17 vs 13 min, p<0.001).

### Primary outcome measure: PSPDR before and after training

**Figure 1:**
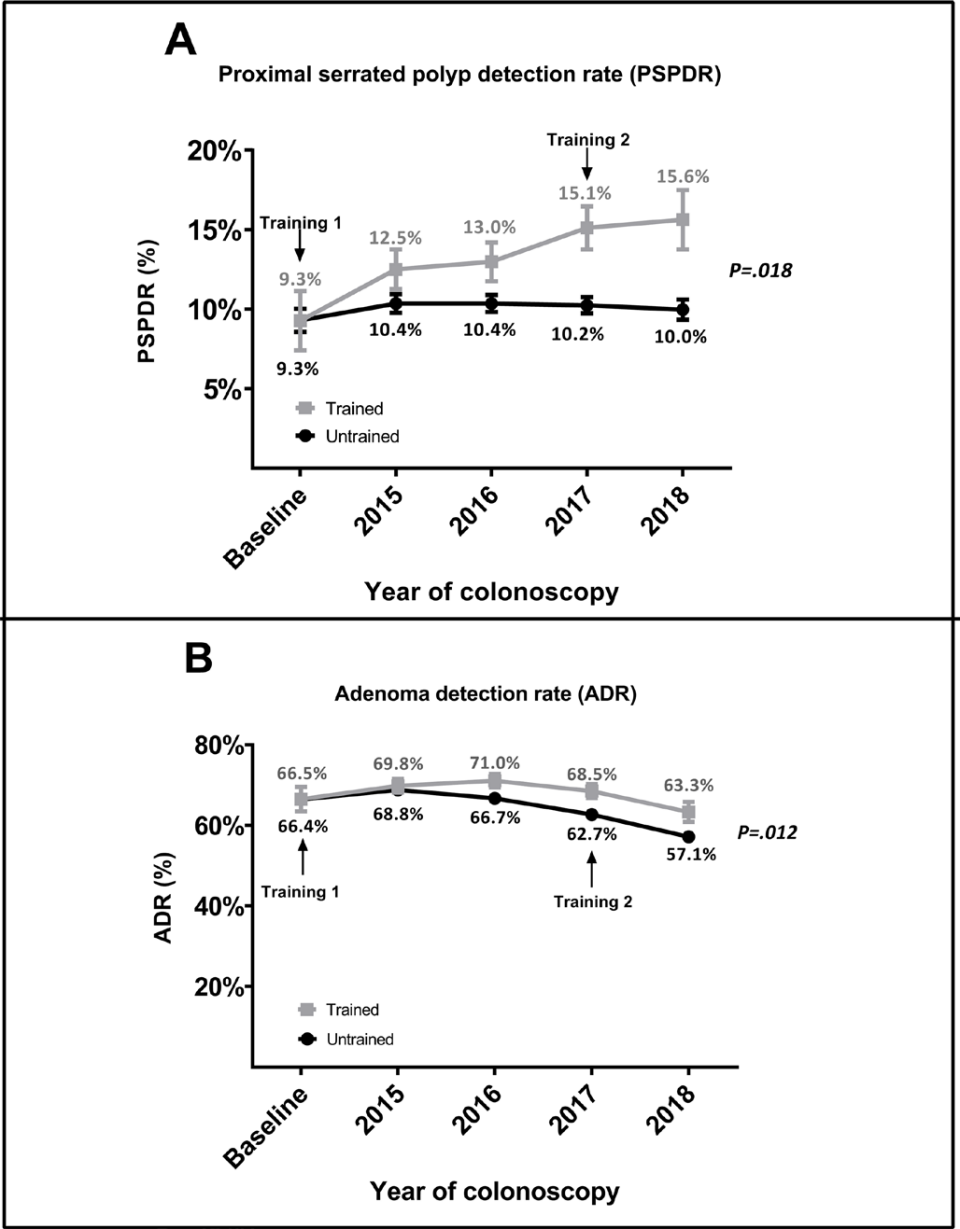
(A) PSPDR before and after training; (B) ADR before and after training. ADR, adenoma detection rate; PSPDR, proximal serrated polyp detection rate.

After receiving their first training, trained endoscopists showed an increase in PSPDR from 9.3% at baseline to 12.5%, 13.0%, 15.1% and 15.6% in 2015, 2016, 2017 and 2018, respectively (table 3 and figure 1A). In contrast, no clear improvement was seen in untrained endoscopists (figure 1A). When stratified per year of follow-up, the difference between trained and untrained endoscopists was non-significant in 2015 (p=0.08) and 2016 (p=0.14), but statistically significant in 2017 (p=0.003) and 2018 (p=0.04). The average PSPDR of trained endoscopists was 35% higher than the PSPDR of untrained endoscopists (13.8% vs 10.2%, p=0.018).

**Table 3:** Post-training detection rates based on colonoscopies performed by trained vs untrained endoscopists

	2015		2016		2017		2018		Overall post-training			
	Trained endoscopists (n=2687)	By untrained endoscopists (n=10 420)	Trained endoscopists (n=2921)	Untrained endoscopists (n=12 295)	Trained endoscopists (n=2700)	Untrained endoscopists (n=13 737)	Trained endoscopists (n=1453)	Untrained endoscopists (n=8515)	Trained endoscopists (n=10 377)	Untrained endoscopists (n=44 967)	OR (95% CI)	P value*
≥1 Proximal SP (=PSPDR), %	12.50%	10%	13%	10%	15%	10%	16%	10%	14%	10%	1.49 (1.07 to 2.07)	**0.018**
≥1 SP	28%	25%	28%	25%	27%	23%	2.70%	21%	28%	24%	1.27 (1.04 to 2.55)	**0.021**
≥1 HP	23%	20%	21.50%	19%	20%	17%	21.50%	16%	22%	18%	1.30 (1.02 to 1.65)	**0.035**
≥1 SSL	8.90%	7.50%	10%	7.80%	11%	7.90%	9.30%	7.40%	9.70%	7.70%	1.30 (1.02 to 1.66)	**0.034**
≥1 TSA	0.70%	1.00%	0.90%	1.00%	0.70%	0.90%	0.60%	0.60%	0.70%	0.90%	0.89 (0.62 to 1.30)	0.58
≥1 Adenoma (=ADR), %	70%	69%	71%	67%	68.50%	63%	63%	57%	68.50%	64%	1.20 (1.04 to 1.38)	**0.012**
≥1 Advanced adenoma	49%	50%	47%	44%	41%	39%	37%	36%	45%	42%	1.08 (0.95 to 1.22)	0.23

Percentages ≥10% are rounded to the nearest integer.

*P values are based on mixed-effects logistic regression analyses, with fixed effects for training and adjusted with random intercepts for type of centre and endoscopist.

ADR, adenoma detection rate; HP, hyperplastic polyp; PSPDR, proximal serrated polyp detection rate; SP, serrated polyp; SSL, sessile serrated lesion; TSA, traditional serrated adenoma.

PSPDR was not associated with the type of centre in which endoscopists worked: compared with academic hospitals, SPs were as frequently detected in general hospitals (p=0.48) and in private hospitals (p=0.45).

When stratifying for SP subtypes, trained endoscopists were more likely to detect both SSLs (9.7% vs 7.7%, OR 1.30 (95% CI 1.02 to 1.66), p=0.034) as well as HPs (22% vs 18%, OR 1.30 (95%CI 1.02 to 1.66), p=0.035, table 3).

#### Sensitivity analyses

Because of the over-representation of academic centres among the group of trained endoscopists, we compared the post-training PSPDR of trained versus untrained endoscopists after exclusion of all colonoscopies performed in academic centres. This yielded similar results to our primary analyses, with an average post-training PSPDR for trained endoscopists of 14.3% vs 10.0% for untrained endoscopists (p=0.004).

Furthermore, we performed sensitivity analyses in which we included the subgroup of endoscopists (n=11) that participated in only one of the training sessions (online supplementary table 1). Based on the 3464 colonoscopies that these endoscopists performed after baseline, they showed a slight increase in PSPDR compared with baseline (10.6% at baseline vs 12.8%), but this was not significantly different compared with untrained endoscopists (OR 1.37, 95% CI 0.91 to 2.06, p=0.13).

10.1136/gutjnl-2019-319804.supp2 Supplementary data

### Effect of first and second training session on trained endoscopists

**Figure 2:**
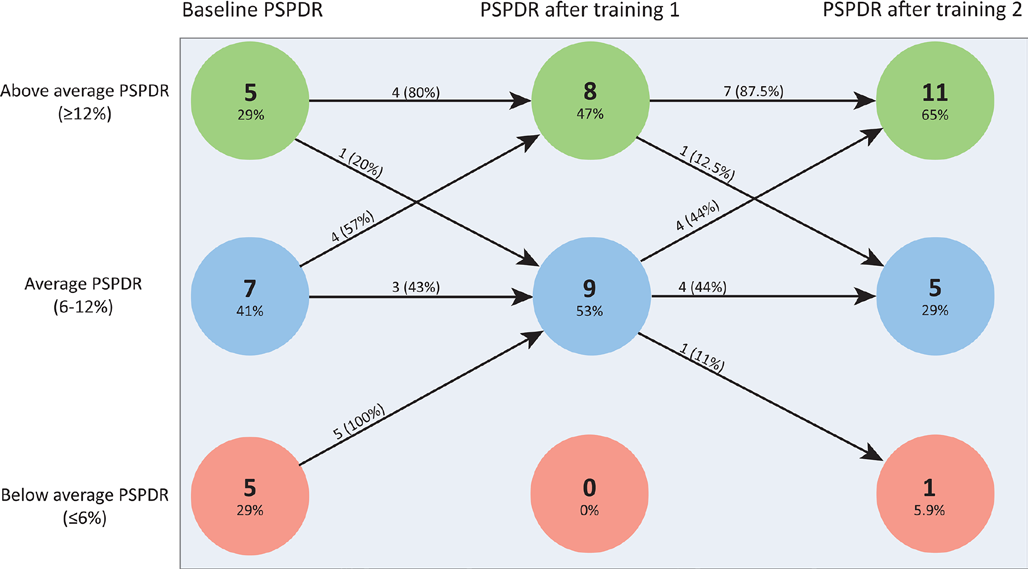
Effect of training sessions 1 and 2 per endoscopist, stratified according to their PSPDR at baseline, after the first training and after the second training. For example, of the seven endoscopists with average PSPDR at baseline, four (57%) moved up to ‘above-average PSPDR’ after training session 1, while three remained in the ‘average PSPDR’ group. PSPDR, proximal serrated polyp detection rate.

**Figure 3:**
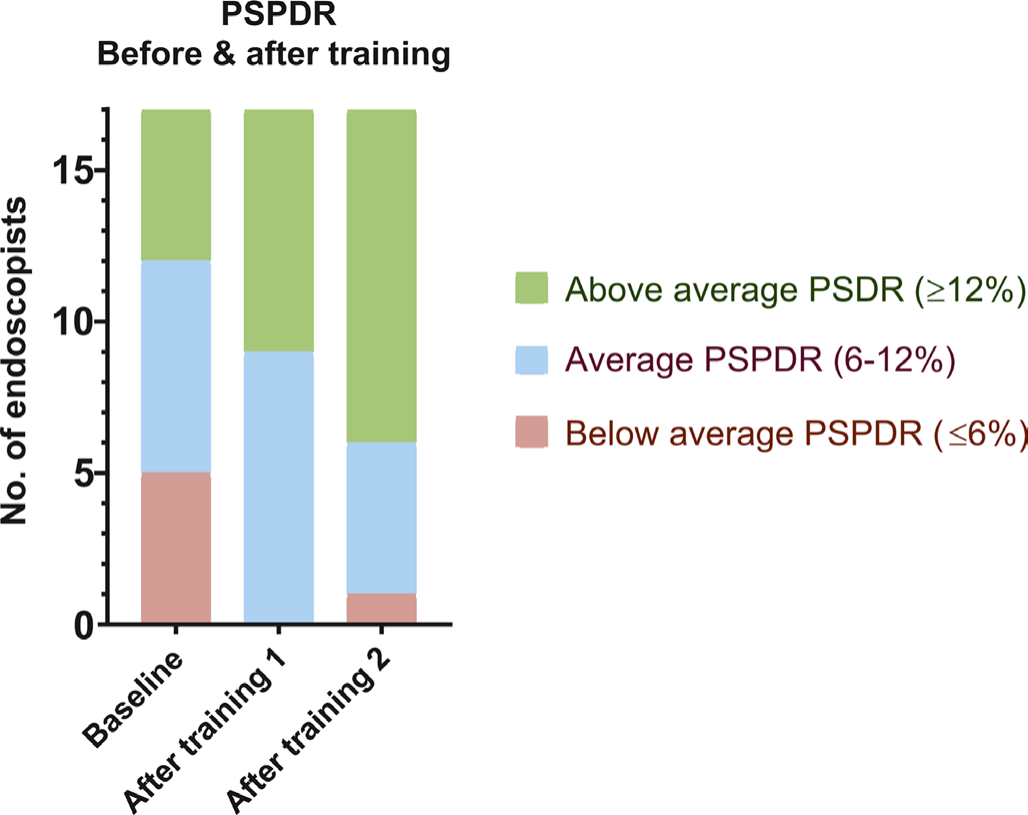
PSPDR before and after training. PSPDR, proximal serrated polyp detection rate.

Based on baseline detection rates, we arbitrarily defined cut-off values to stratify endoscopists into below-average PSPDR (≤6%), average PSPDR (6%–12%) and above-average PSPDR (≥12%) (figures 2 and 3).

#### Endoscopists with below-average PSPDR at baseline (n=5)

All five endoscopists with below-average PSPDR at baseline improved after the first training session. After the second training session, one endoscopist showed additional improvement and fell in the range of above-average, while one fell back to the below-average range after training session 2. The other three remained in the range of average PSPDR after training session 2.

#### Endoscopists with average PSPDR at baseline (n=7)

Of the seven endoscopists with average PSPDR at baseline, four improved to above-average PSPDR after training session 1. The second training session resulted in improvement of the remaining three, who also moved to above average PSPDR. In other words, all endoscopists with average baseline PSPDR had above-average PSPDR after the second training session.

#### Endoscopists with above-average PSPDR at baseline (n=5)

Of the five endoscopists with an above-average PSPDR at baseline, four remained in the above-average range after training session 1, while one fell back to average PSPDR. After training session 2, one additional endoscopists fell back to average PSPDR. In other words, three of the five endoscopists with above-average PSPDR at baseline also had above-average PSPDR after the second training session, while two fell back to average PSPDR.

### Secondary outcome measure: ADR before and after training

The ADR of trained and untrained endoscopists did not differ at baseline (66.5% vs 66.4%, respectively). Overall, trained endoscopist had an average post-training ADR of 68.5%, while untrained endoscopists had an average ADR of 64.1%, corresponding to a relative difference of 6.9%, and an OR of 1.2 (95% CI 1.04 to 1.38, p=0.012) for the detection of one or more adenomas (table 3 and figure 1B). During follow-up, the ADR of both trained and untrained endoscopists initially increased before decreasing in 2017 and 2018 to below baseline detection rates: the ADR of trained endoscopists slightly increased to 69.8% and 71.0% in 2015 and 2016, and then dropped to 68.5% and 63.3% in 2017 and 2018, respectively. Comparably, the ADR of untrained endoscopists slightly increased to 68.8% in 2015, before falling to 66.7%, 62.7% and 57.1% in 2016, 2017 and 2018, respectively (table 3 and figure 1B).

## Discussion

In this large prospective trial, we demonstrate that a simple educational intervention was associated with substantial and sustained increase in PSPDR during colonoscopy within a homogeneous FIT-positive screening population. Considering that these lesions are held responsible for a disproportionately high number of interval carcinomas,^8 16 30 31^ our intervention might ultimately lead to a significant reduction in PCCRCs within FIT-based screening programmes. In addition, we demonstrate that the most prominent effect was achieved in endoscopists that had a below-average PSPDR at baseline (figure 2), suggesting this intervention should be mainly targeted at the low-performers.

Interpreting our findings, lack of *awareness* about SPs might be one of the most important contributors to the highly variable (and often suboptimal) SP detection as described in previous studies.^11 12^ After all, the content of our training sessions mostly focused on *why* SPs should be detected and resected, rather than how this should be done. This might be due to the fact that the contribution of SPs to CRC has only gradually come into focus over the past two decades.^5 9 32–35^ Many endoscopists were trained in an era in which adenomas were considered the sole precursors to CRC, or were trained *by* endoscopists that were trained in this pre-SP era. Indeed, a recent large multicentre study by Crockett *et al* demonstrated that endoscopists with fewer years of practice detected significantly more SPs than their older colleagues.^36^

Apart from the increased SP detection, we saw an interesting time-trend in the ADR: after initially increasing, both groups showed a decreasing ADR in 2017 and 2018 (figure 1B). This trend was in line with an overall slow decrease in ADR in our national screening programme. This can be attributed to three factors related to the implementation of the Dutch biennial FIT-based screening programme from 2014 onwards: an increase of the iFOBT cut-off from 88 to 275 ng/mL in 2014. First, the initial increase in ADR was most likely a result of the increase of the cut-off of the iFOBT from 88 to 275 ng/mL.^37^ The subsequent decrease in ADR can be attributed to the decreasing age of invitees from 2015 onwards, which was in turn a result of the phased invitation of screenees from 2014 to 2019 (online supplementary figure 1). Lastly, the screening programme consists of biennial FIT. As a result, the proportion of participants in their second-round or third test-round grew from 0% in 2014 and 2015, to 30% in 2016 and 53% in 2017.^37–40^ In line with literature,^41–44^ the second-round/third-round screenees had a lower incidence of CRC, advanced adenomas and non-advanced adenomas, which reflects the lower positive predictive value of FIT after multiple test-rounds. Regardless of this overall trend, endoscopists that received our SP detection training seemed to have a slightly higher ADR than endoscopists that did not receive our training (p=0.012). Although statistically significant, the clinical relevance of this minor difference in ADR is questionable. The reason for this difference in ADR between intervention and control group is unclear, but could potentially relate to the longer withdrawal time in the intervention group. Alternatively, more thorough mucosal inspection in search of SPs in the intervention group might have also resulted in higher detection of adenomas.

10.1136/gutjnl-2019-319804.supp3 Supplementary data

To interpret our results, it should be questioned whether these background developments in the population screening programme might have also affected our study outcomes. This does not seem to be the case because these developments have equally affected the intervention and control arm of our study.

Our study has several strengths. First of all, this is the first study focusing on educational interventions to improve SP detection. Such targeted interventions are needed, as Racho *et al* showed that training focused on improving ADR detection had no effect whatsoever on SP detection, despite its long-lasting beneficial effect on ADR.^22^ Second, we had the opportunity to perform our study within the Dutch FIT-based screening programme. This allowed us to include a large number of endoscopists and colonoscopies. Our cohort of 17 intervention and 100 control endoscopists can be considered large compared with the well-known EQUIP trial which assessed the effectivity of a training which aimed to improve ADR, in which eight and seven endoscopists were randomised into the intervention and control arm, respectively.^19^ Equally important, nesting our study in the Dutch screening programme provided us with a prospectively collected database of a homogeneous group of patients with high-quality colonoscopies performed by accredited endoscopists. Quality indicators (ie, bowel preparation, caecum intubation and inspection time) were each met in over 95% of colonoscopies (table 1). Third, we included almost 5 years of prospective follow-up data, which allowed us to assess the long-term impact of our educational intervention. Last, the histopathological assessment, especially the SP subclassification, can be considered high quality as well since all pathologists received online training in order to optimise correct SP diagnosis. This online training module was shown to be very effective.^45^

Nevertheless, several alternative explanations for the improved PSPDR have to be considered as well. First of all, trained endoscopists were aware of the fact that they participated in this study, while the untrained endoscopists were not. As such, the Hawthorne effect, which describes an individual’s change in behaviour due to awareness of being observed,^46^ might have positively influenced the PSPDR of trained, but not of untrained endoscopists. However, we believe this effect has not played a major role in our study. First, the awareness of being monitored among trained endoscopists was kept to an absolute minimum in order to minimise interference of the Hawthorne effect. The background monitoring of PSPDR was only communicated verbally during both training sessions. Aside from these brief occasions in 2014 and 2017, no communication existed between the researchers and the endoscopists, and the endoscopists were not reminded in any other way about our ongoing study. It therefore seems likely that our ongoing study was not on top of the mind of participating endoscopists during their daily work in the 5 years of follow-up. Moreover, *all* Dutch endoscopists are aware that they are being monitored as part of standard care, since they continuously have to meet several minimal quality requirements (eg, ADR ≥30%).^25^ Since endoscopists face penalties when they fail to reach such quality parameters (eg, withdrawal of accreditation), our monitoring was probably perceived as far less urgent and thus received less attention from endoscopists than the ongoing quality assurance parameters they were confronted with in daily practice. Nevertheless, it seems advisable that future similar studies make the control arm aware of the fact that they are monitored too, in order to reduce any potential influence of the Hawthorne effect.

Furthermore, our study was not randomised, and therefore we cannot rule out some degree of selection bias. Baseline differences between the trained and untrained endoscopists might have played a role in the increased detection rates we observed in the trained group. First of all, trained endoscopists more often operated in academic centres (12% vs 24%, p<0.001). However, we accounted for this by including type of centre in our multivariable model. In addition, our regression analyses did not show any association between type of centre and PSPDR, neither in trained or untrained endoscopists. Moreover, we ran separate sensitivity analyses in which we excluded colonoscopies in academic centres, which yielded similar results. A second baseline difference concerns the mucosal inspection time (16 vs 13 min, p<0.001), suggesting trained endoscopists already took slightly more time for mucosal inspection than untrained endoscopists before their first training. However, although a prolonged withdrawal time has been linked to superior ADR,^47–51^ both groups exceeded withdrawal times of 10 min. A study by Lee *et al* demonstrated that the additional benefit of withdrawal times beyond 10 min is questionable, and minimal at best.^51^ We therefore consider it unlikely that this baseline difference represents a clinically relevant bias.

We cannot rule out that these or other baseline differences might have influenced our results. Most importantly, however, our baseline comparison of detection rates shows that across all polyp subtypes, detection rates of trained and untrained endoscopists were virtually identical (table 2). This strongly suggests that trained and untrained endoscopists were indeed ‘equal’ at baseline.

Several other limitations have to be acknowledged as well. We chose to include endoscopists that attended both training sessions only, which meant several had to be excluded due to absence during either training session. Since attendance was voluntary, this might have led to selection of more devoted or motivated endoscopists. Considering that the effect of our training strongly relies on endoscopists’ motivation to pay extra attention to serrated lesions, this selection might have caused an overestimation of our outcome parameters. However, this potential selection bias seems less plausible taking into account our sensitivity analysis, in which endoscopists that only participated in one of the two training sessions also showed an increase in PSPDR from 10.6% to 12.8%, although this was not significantly different from the untrained group (p=0.13), possibly due to the small subgroup sample size (3464 colonoscopies in 11 endoscopists).

Another limitation was the scarcity of endoscopist characteristics we could include in our analysis. It would have been very informative to assess whether characteristics like age, years of experience or gender might predict the effect of our intervention. Unfortunately, such parameters could not be provided by the RIVM because their database did not contain this information. Finally, our secondary analyses to assess which endoscopists benefited most from our interventions required us to split up our intervention group in three subgroups. The resulting subgroups are quite small (ie, five, seven and five endoscopists in the below-average, average and above-average groups, respectively). These subgroup analyses should therefore be interpreted with caution and warrant validation in future larger studies.

Our results would ideally be interpreted in the light of established, evidence-based targets for PSPDR to optimise PCCRC prevention, similar to the studies that were used to guide ADR targets. For example, Kaminski *et al* demonstrated that an ADR of at least 20% was associated with a much lower risk of PCCRC,^17^ while Corley *et al* later demonstrated that every 1% increase in ADR was associated with a 3% decrease in PCCRC incidence.^18^ Such data have been invaluable in determining clinically meaningful ADR targets. Unfortunately, no such data have been published on SP detection rates, and therefore it is much more difficult to determine clinically meaningful PSPDR targets at this moment. We do know, however, that PCCRCs disproportionately often bear typical molecular and clinical characteristics of the serrated pathway, and therefore it is generally believed that missing SPs is an important factor in PCCRC development.^5 8 16 52^ This suggests that improvement of SP detection would result in a decline in PCCRC incidence. Indeed, recently the British Society for Gastroenterology even suggested that improvement of SP detection might have a greater impact on PCCRC incidence than improvement of ADR.^8^ In this light, and in the absence of established thresholds, it seems safe to assume that any improvement in PSPDR might be clinically relevant. Future studies will hopefully help to establish evidence-based PSPDR thresholds to further guide the implementation and targeting of quality improvement interventions such as ours.

In conclusion, our results show that improvement of SP detection can be achieved relatively easily by a simple educational intervention, which consists of two 45 min training sessions which are held 3 years apart. Giving SPs a more prominent place in current endoscopy programme could potentially lead to improved CRC prevention. Endoscopists with a low PSPDR at baseline benefited most from our intervention, which supports introduction of continuous PSPDR monitoring in order to identify and target low performers and provide additional training to them.
