# Supplementary material for: Substantial and sustained improvement of serrated polyp detection after a simple educational intervention: results from a prospective controlled trial
Source: Gut. 2020 Mar 5;69(12):2150–8. doi: 10.1136/gutjnl-2019-319804 (PMC7677479; doi:10.1136/gutjnl-2019-319804)
Supplement: Supplementary data [file gutjnl-2019-319804supp001.pdf]

Serrated polyp awareness training

Supplementary material to manuscript entitled:

"Substantial and sustained improvement of serrated polyp detection after a simple educational intervention – Results from a prospective controlled trial"

Arne GC Bleijenberg, Monique E. van Leerdam, Marloes Bigirwamungu-Bargeman, Jan Jacob Koornstra, Yasmijn J van Herwaarden, Manon C.W. Spaander, Silvia Sanduleanu, Barbara Bastiaansen, Erik J. Schoon, Niels van Lelyveld, Evelien Dekker, Joep EG IJspeert

Optimizing the detection of colonic serrated polyps

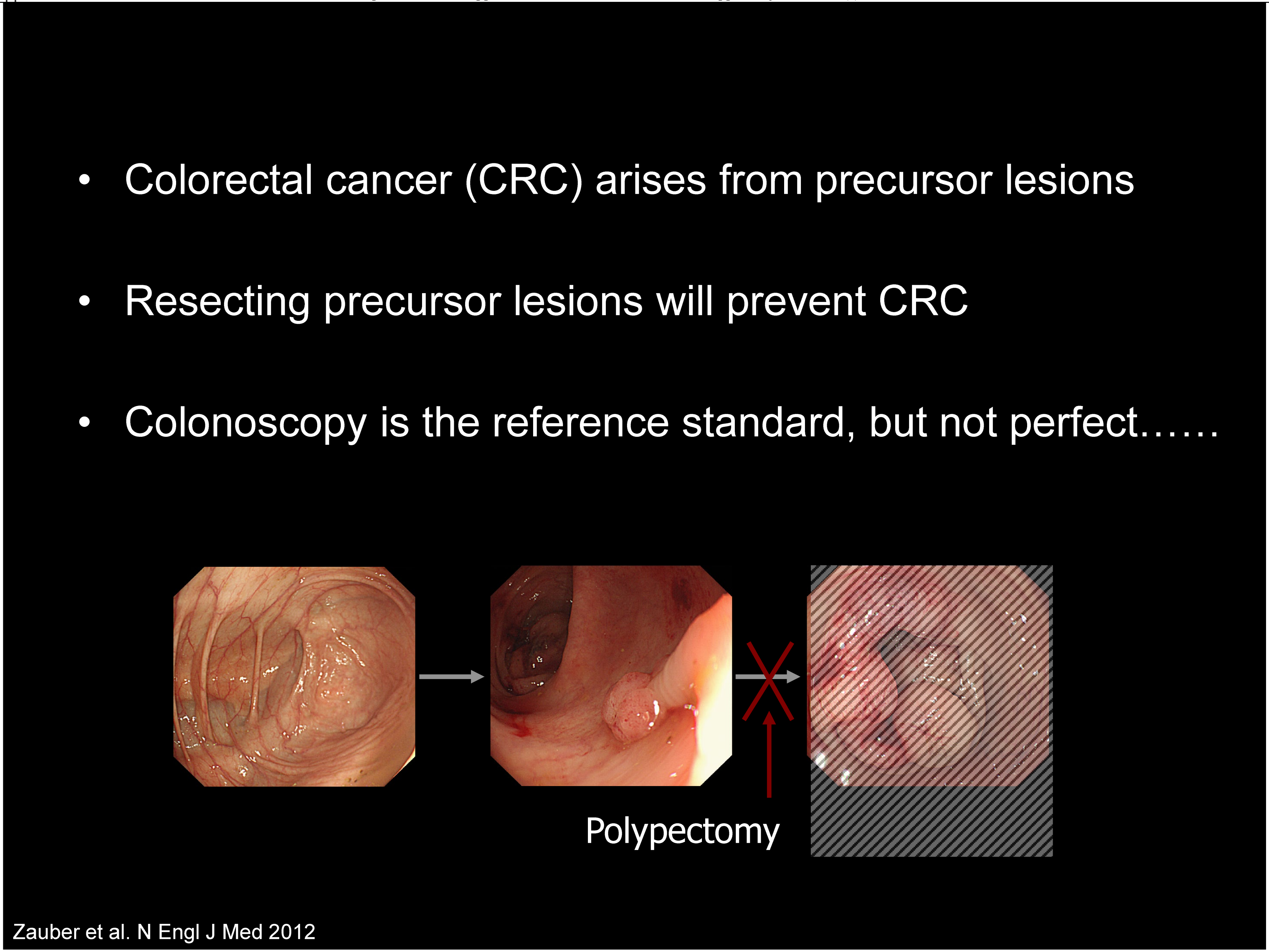
• Colorectal cancer (CRC) arises from precursor lesions
• Resecting precursor lesions will prevent CRC
• Colonoscopy is the reference standard, but not perfect……
Polypectomy
Zauber et al. N Engl J Med 2012

Colonoscopy protects for left-sided..

Table 3. **Results of Primary Analysis: Odds Ratio for the Association Between Colonoscopy and Colorectal Cancer Death***

Model	Odds Ratio (95% CI)			
	All Cancer	Right-Sided Cancer	Left-Sided Cancer	Undefined Site of Cancer
**Attempted colonoscopy**				
None	1.00	1.00	1.00	1.00
Any	0.69 (0.63–0.74)	1.07 (0.94–1.21)	0.39 (0.34–0.45)	0.90 (0.75–1.08)
**Completeness of colonoscopy**				
None	1.00	1.00	1.00	1.00
Complete	0.63 (0.57–0.69)	0.99 (0.86–1.14)	0.33 (0.28–0.39)	0.90 (0.73–1.10)
Incomplete	0.91 (0.78–1.07)	1.35 (1.07–1.69)	0.63 (0.49–0.81)	0.91 (0.61–1.35)

* Conditional logistic regression, adjusted for Charlson Comorbidity Index score.

.. but not for right-sided colorectal cancer

Baxter et al. Ann Int Med 2009

Reasons for right-sided interval cancers:

- Inadequate quality of coloscopy, e.g. no cecal intubation or insufficient bowel preparation
- Inadequate detection and resection of serrated polyps (SPs)

Table 2. Molecular characteristics of interval vs. non-interval cancers

	Interval	Non-interval	*P* value
CIMP[a]			
Positive	31 (57%)	33 (33%)	0.004
Negative	23 (43%)	75 (66%)	

Arain et al. AJGE 2010

Serrated polyps

	Shape	Mean Size	Prevalence	Location	Pre-cancerous
HP	Flat, sessile	Small, often ≤ 5 mm	Very common	Left colon	No
SSA/P	Flat, sessile	Larger than HP[a]	Common[b]	Right colon	Yes
TSA	Sessile, pedunculated	Larger than HP	Rare	Left colon	Yes

Rex et al. Am J Gastroenterol 2012; 107:1315–1329

Prevalence of SPs

% of asymptomatic patients with at least 1 SP:

HP	23.8 %
SSA/P	4.8%
SSA/P +D	1.5%
TSA	0.1%

	Prevalence
	Total (n = 1426)[1]
**At least 1 serrated polyp, n (%)**	388 (27.2)
Hyperplastic polyp	339 (23.8)
SSA/P	68 (4.8)
TSA	1 (0.1)
**At least 1 proximal serrated polyp, n (%)**	174 (12.2)
Proximal hyperplastic polyp	127 (8.9)
Proximal SSA/P	51 (3.6)
Proximal TSA	0.0 (0.0)
**At least 1 serrated polyp ≥ 10 mm, n (%)**	37 (2.6)
Hyperplastic polyp ≥ 10 mm	22 (1.5)
SSA/P ≥ 10 mm	16 (1.1)
TSA ≥ 10 mm	0.0 (0.0)
**At least 1 serrated polyp with dysplasia, n (%)**	22 (1.5)
SSA/P with dysplasia	21 (1.5)
TSA with dysplasia	1 (0.1)

Hazewinkel et al. Endoscopy 2014

Prevalence of SSA/P

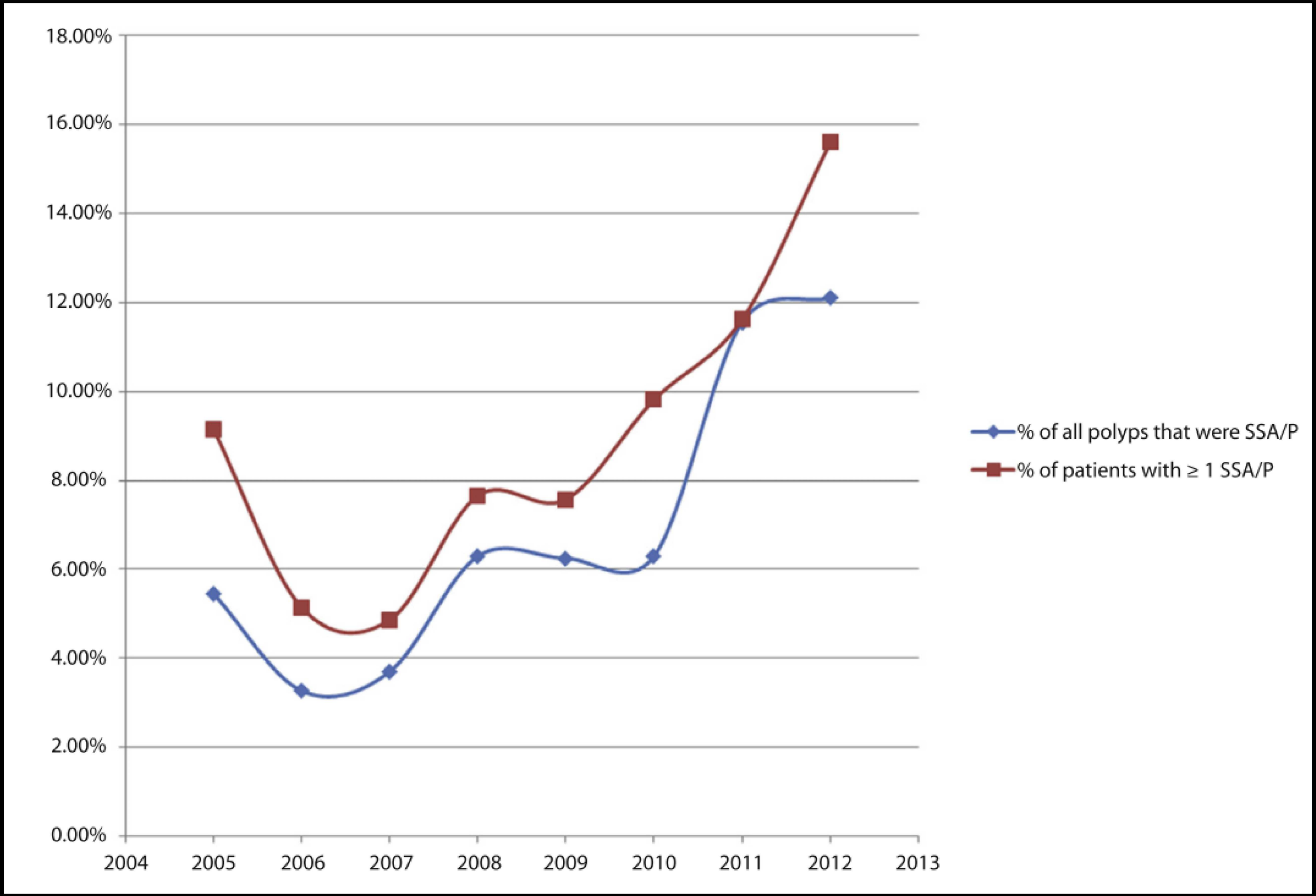

Abdeljawad et al. GIE 2014

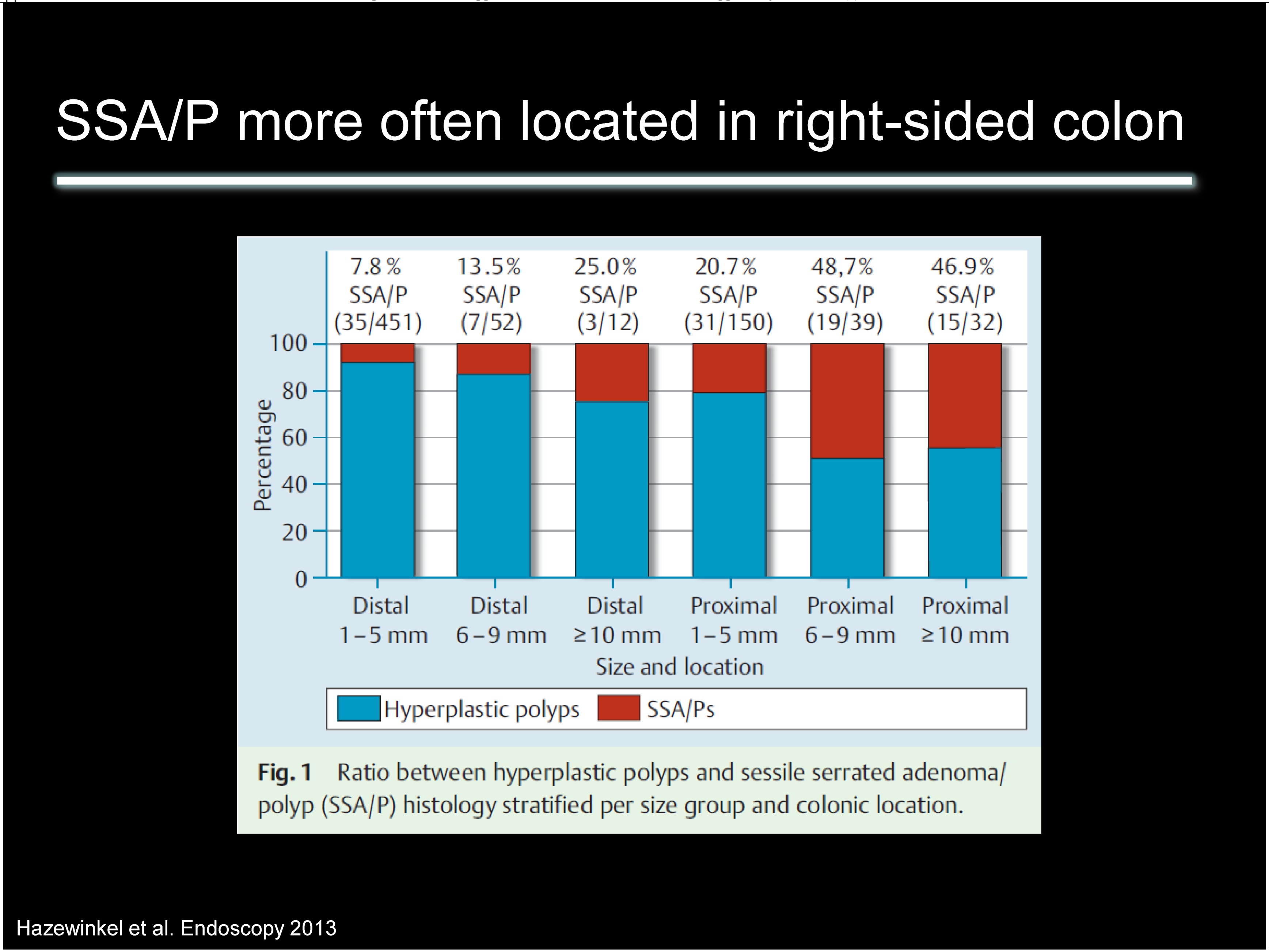
SSA/P more often located in right-sided colon
7.8 % SSA/P (35/451)
13.5% SSA/P (7/52)
25.0% SSA/P (3/12)
20.7% SSA/P (31/150)
48,7% SSA/P (19/39)
46.9% SSA/P (15/32)
Percentage
100
80
60
40
20
0
Distal 1–5 mm
Distal 6–9 mm
Distal ≥10 mm
Proximal 1–5 mm
Proximal 6–9 mm
Proximal ≥10 mm
Size and location
Hyperplastic polyps
SSA/Ps
Fig. 1 Ratio between hyperplastic polyps and sessile serrated adenoma/polyp (SSA/P) histology stratified per size group and colonic location.
Hazewinkel et al. Endoscopy 2013

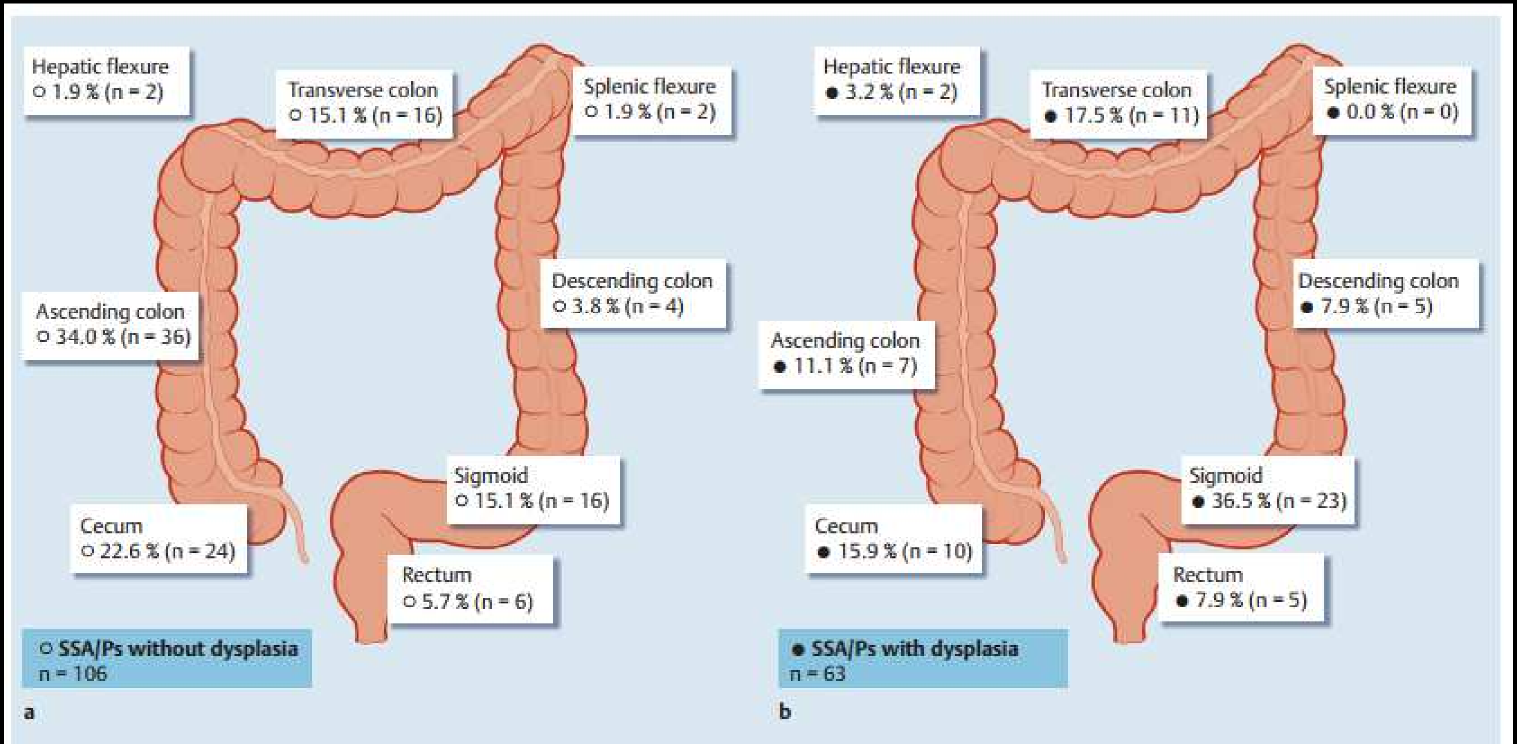

BUT…

SSA/P with dysplasia located throughout the colon??

Bouwens et al. Endoscopy 2014

BMJ Publishing Group Limited (BMJ) disclaims all liability and responsibility arising from any reliance placed on this supplemental material which has been supplied by the author(s)

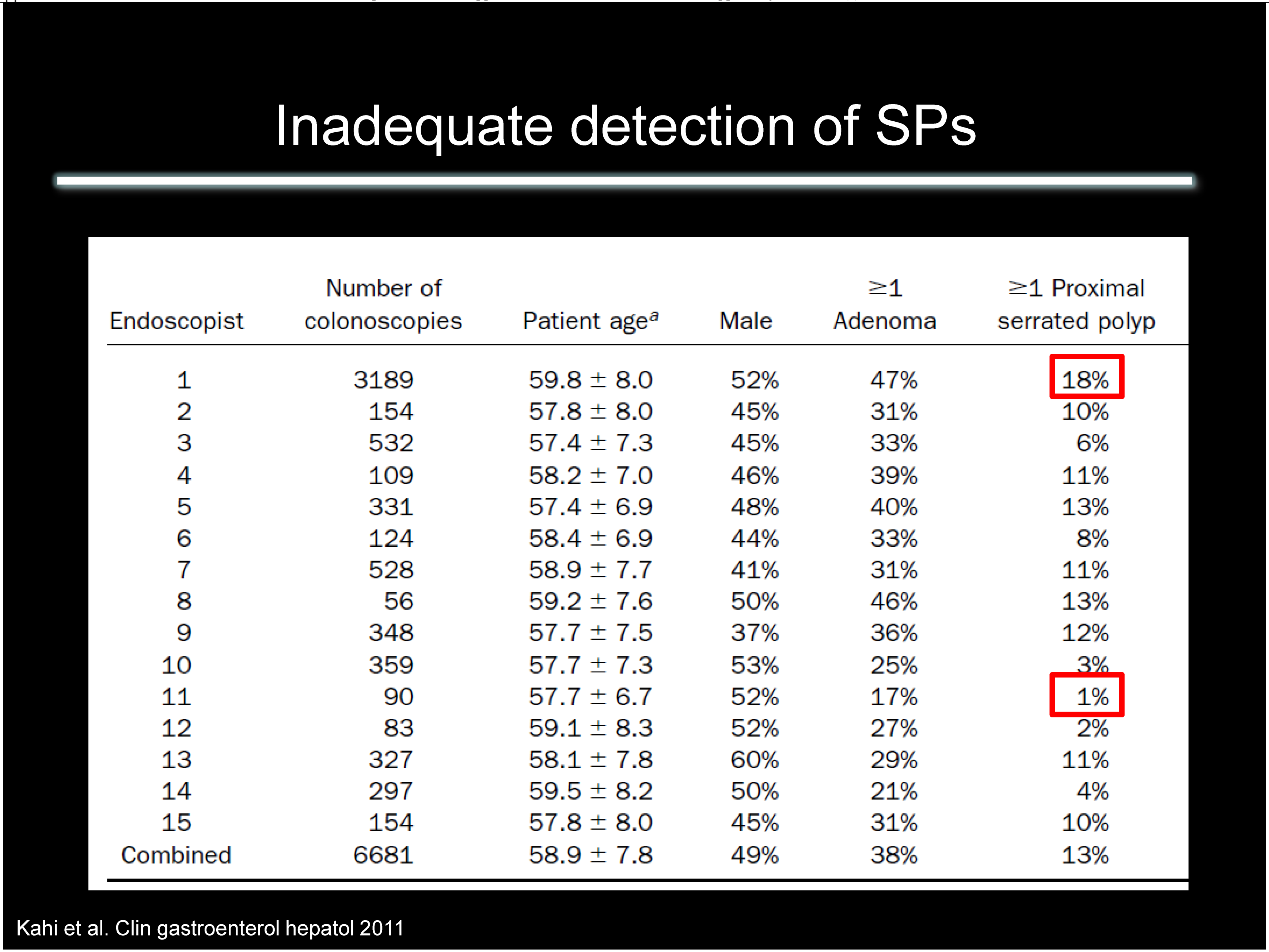

Inadequate detection of SPs

Endoscopist	Number of colonoscopies	Patient age[a]	Male	≥1 Adenoma	≥1 Proximal serrated polyp
1	3189	59.8 ± 8.0	52%	47%	18%
2	154	57.8 ± 8.0	45%	31%	10%
3	532	57.4 ± 7.3	45%	33%	6%
4	109	58.2 ± 7.0	46%	39%	11%
5	331	57.4 ± 6.9	48%	40%	13%
6	124	58.4 ± 6.9	44%	33%	8%
7	528	58.9 ± 7.7	41%	31%	11%
8	56	59.2 ± 7.6	50%	46%	13%
9	348	57.7 ± 7.5	37%	36%	12%
10	359	57.7 ± 7.3	53%	25%	3%
11	90	57.7 ± 6.7	52%	17%	1%
12	83	59.1 ± 8.3	52%	27%	2%
13	327	58.1 ± 7.8	60%	29%	11%
14	297	59.5 ± 8.2	50%	21%	4%
15	154	57.8 ± 8.0	45%	31%	10%
Combined	6681	58.9 ± 7.8	49%	38%	13%

Kahi et al. Clin gastroenterol hepatol 2011

Inadequate detection of SPs

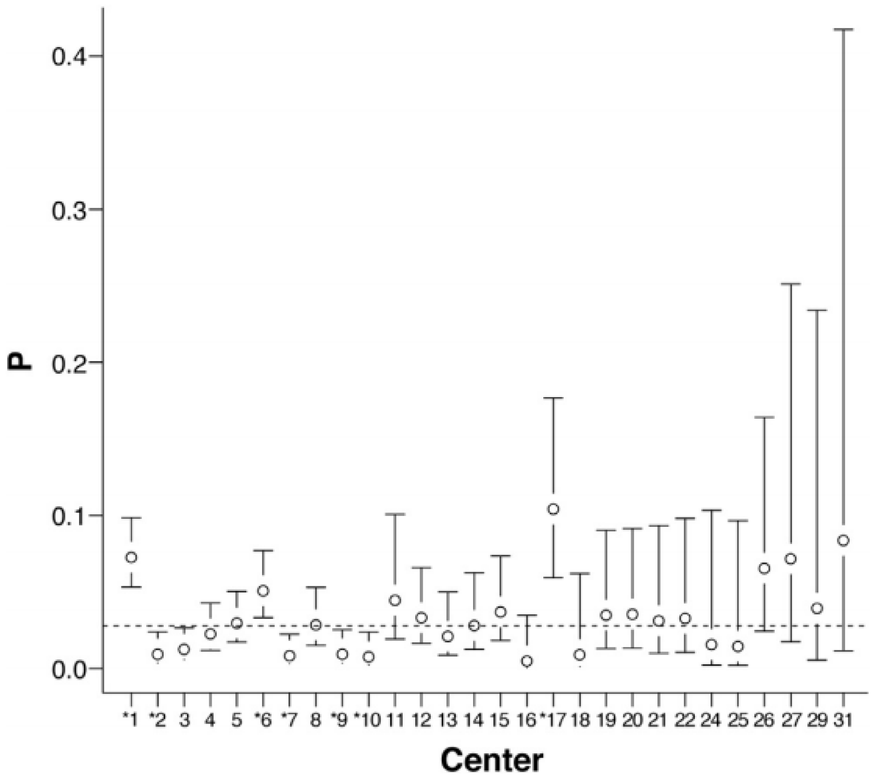

Figure 1. Prevalence rates of proximal serrated lesions in 31 centers. The vertical axis (*P*) is the fraction of patients with 1 or more proximal serrated lesions. The average detection rate of 2.8% is designated by the *hashed line*.

Payne et al. Clin Gastro and Hepatol 2014

BMJ Publishing Group Limited (BMJ) disclaims all liability and responsibility arising from any reliance placed on this supplemental material which has been supplied by the author(s)

How to improve serrated polyp detection?

- Improve awareness of malignant potential of SPs
- Improve knowledge of the subtle SP features: know what you are looking for!
- Ensure a high quality colonoscopy
- Use advanced imaging techniques for improved surveillance in case of uncertainty

Malignant potential of SPs

± 20-30% of CRC arises from SPs

Pathogenesis via serrated neoplasia pathway

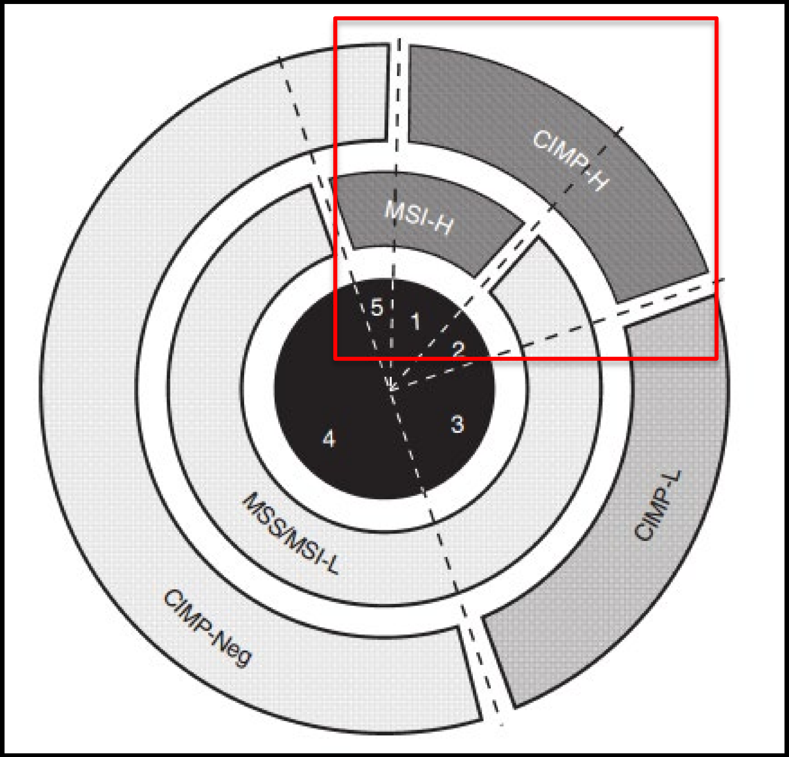

Jass et al. Histopathology 2006

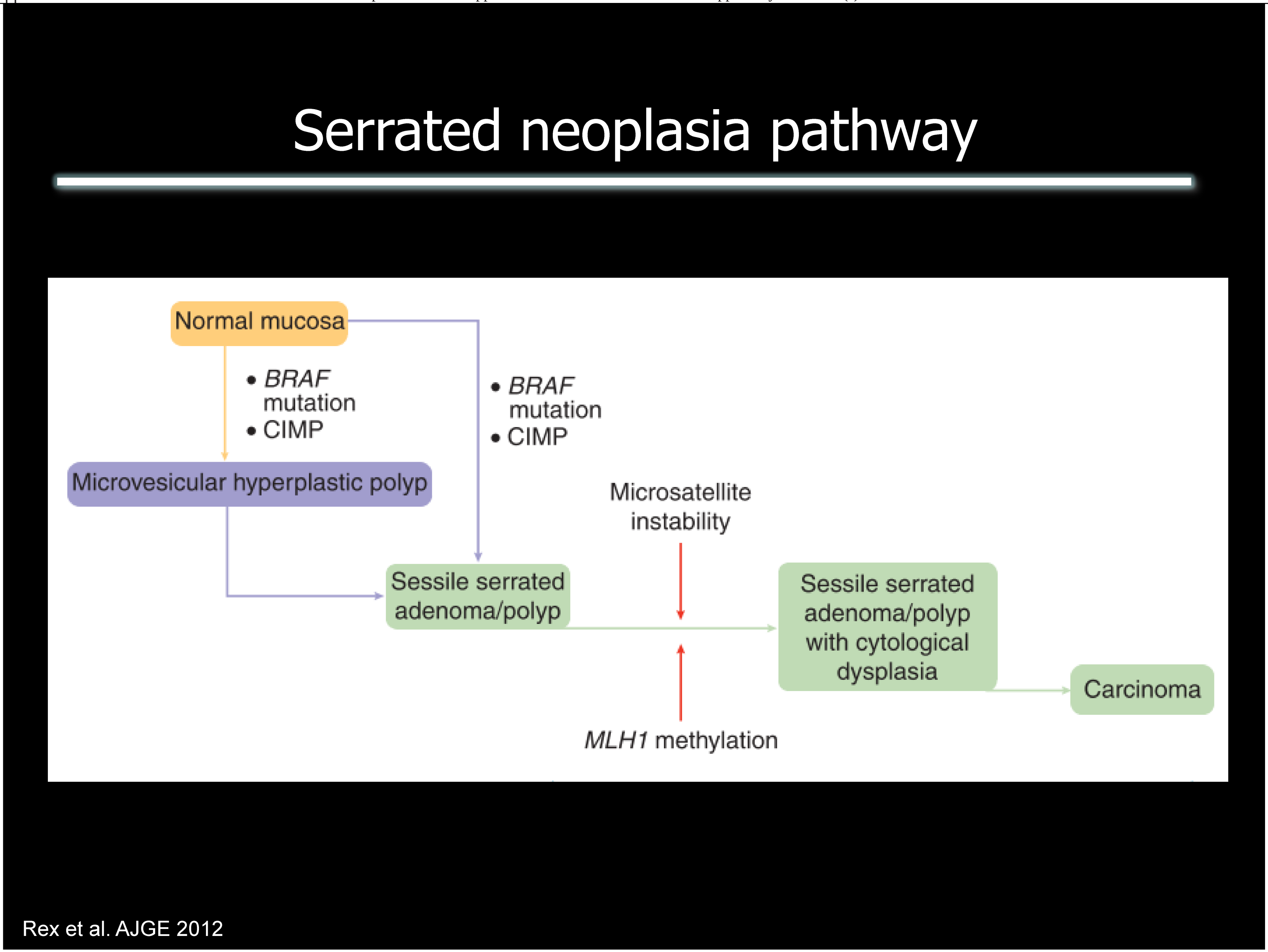
Serrated neoplasia pathway
Normal mucosa
• *BRAF* mutation
• CIMP
• *BRAF* mutation
• CIMP
Microvesicular hyperplastic polyp
Microsatellite instability
Sessile serrated adenoma/polyp
Sessile serrated adenoma/polyp with cytological dysplasia
Carcinoma
MLH1 methylation
Rex et al. AJGE 2012

Why are serrated polyps easily missed?

Subtle features of SPs:

- Flat or sessile morphology
- Same color as mucosal wall
- Vague borders

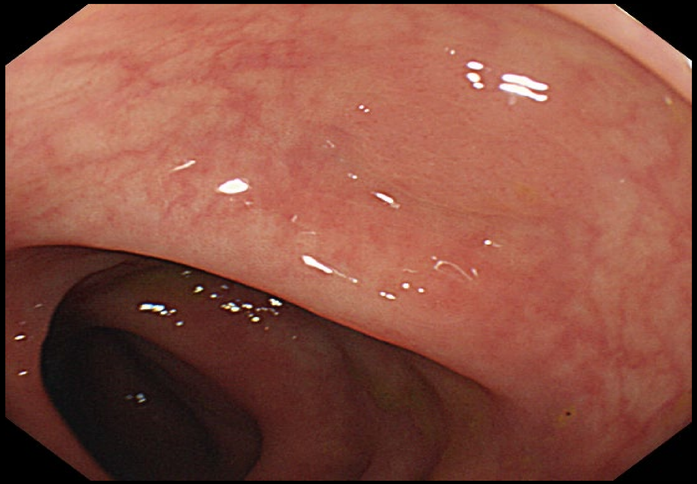

Knowledge of SP features will improve detection!

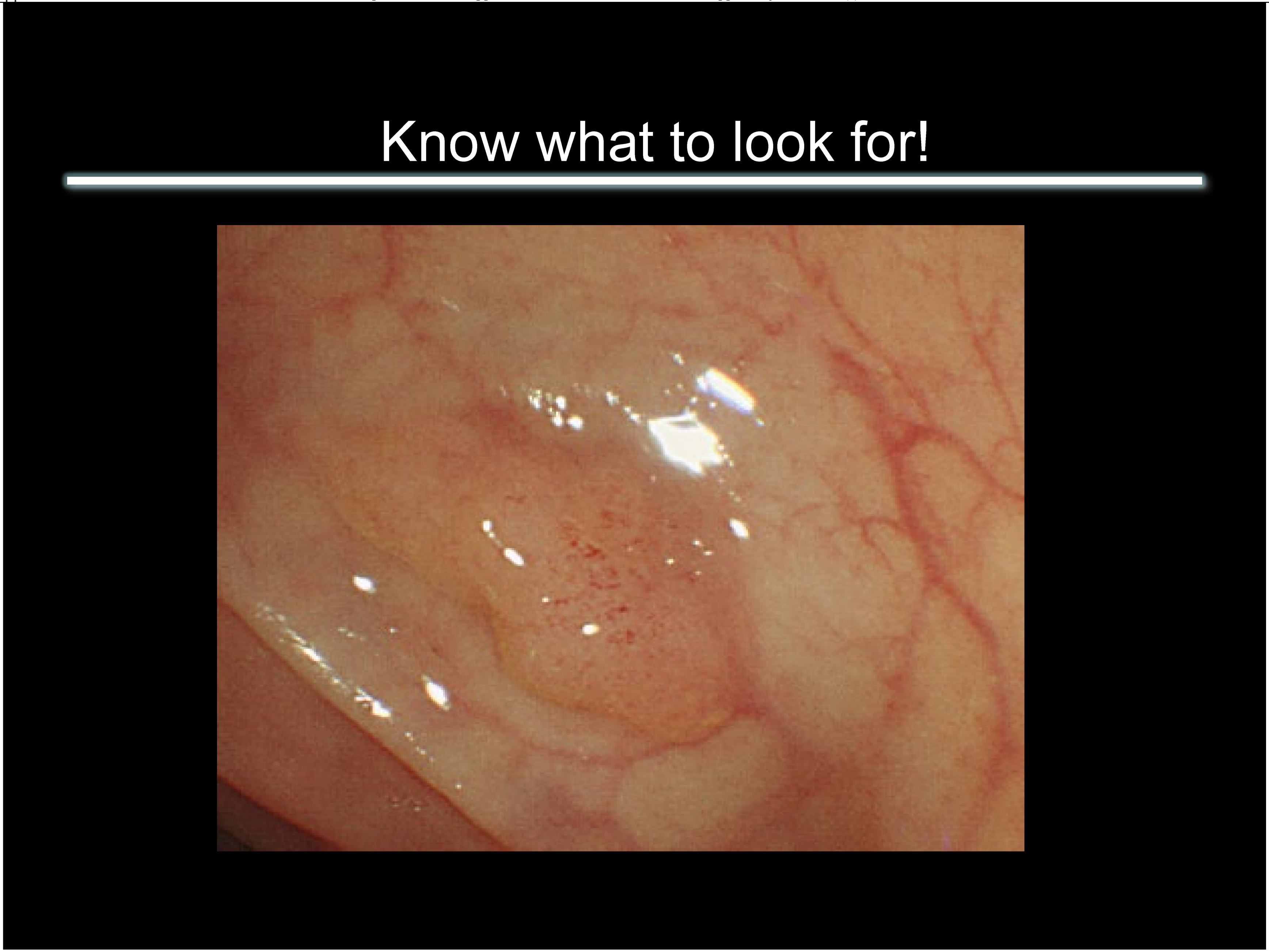
Know what to look for!

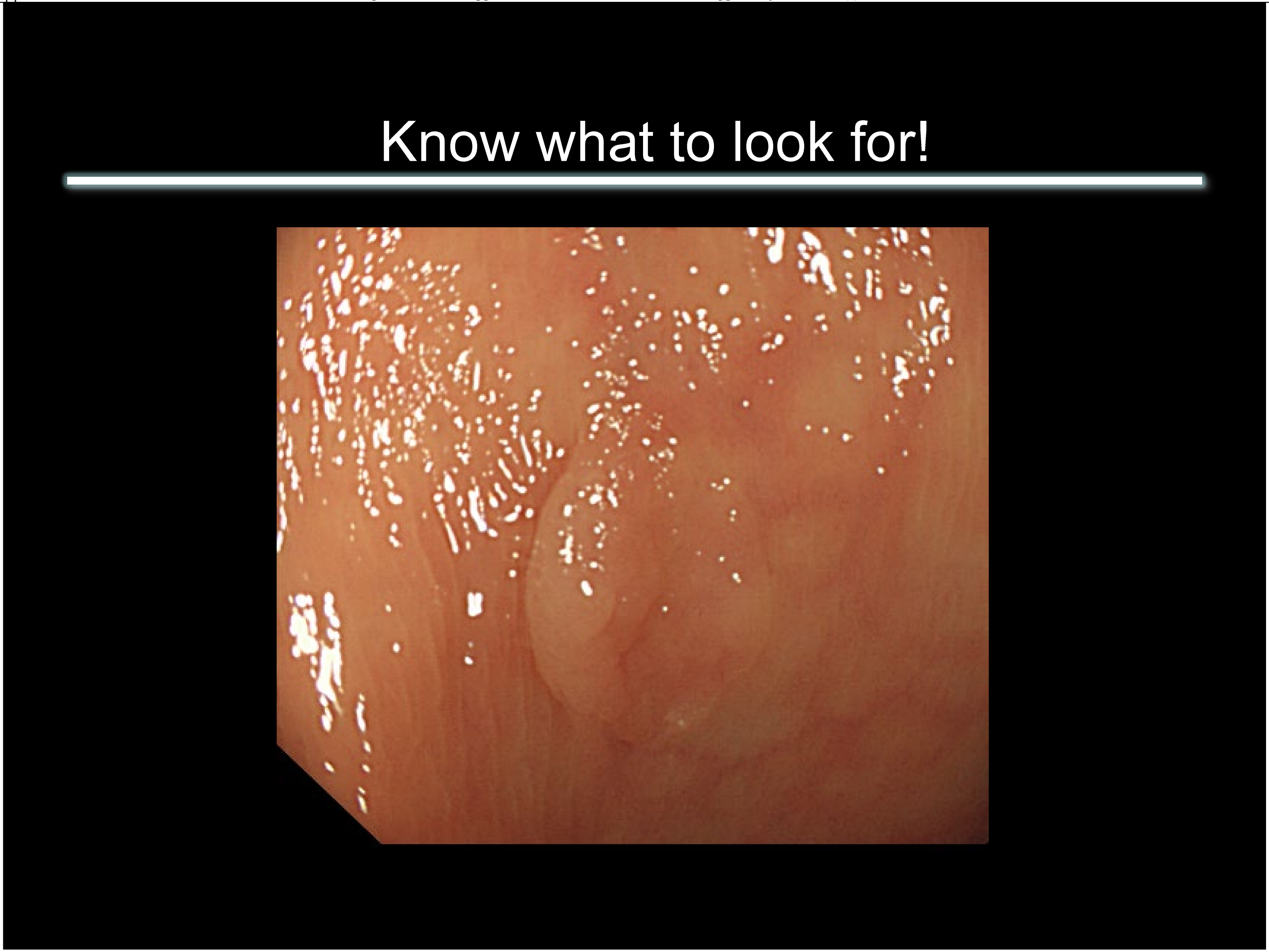
Know what to look for!

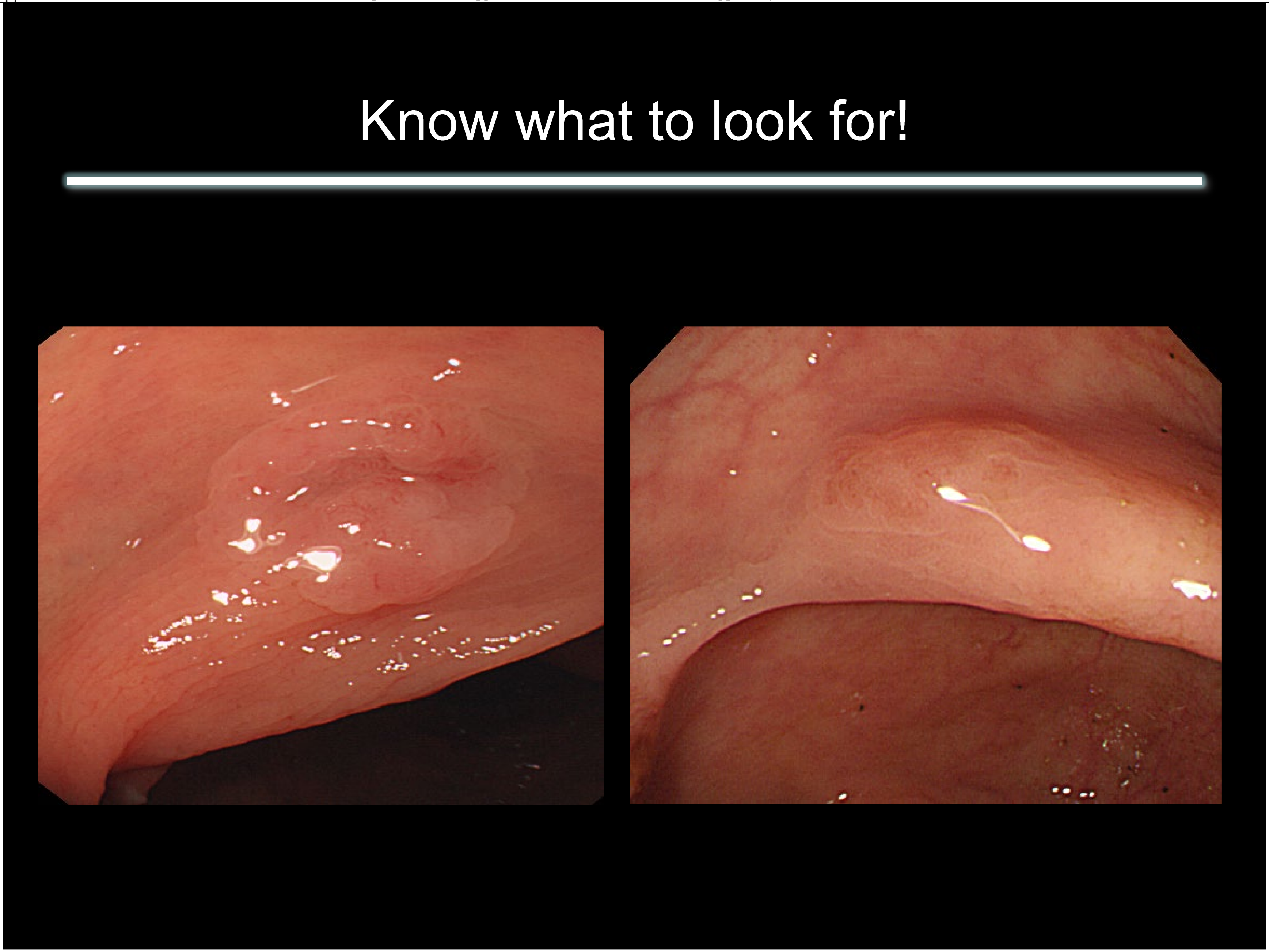
Know what to look for!

Optimizing the characterization of colonic serrated polyps

Characterizing SSA/P

	SSA/P	HP/Adenoma
Surface	Clouded	Smooth
Borders	Indistinct (vague)	Sharp demarcated
Shape	Irregular	Symmetric
Crypts	Dark spots inside the crypts	No dark spots inside the crypts

Hazewinkel et al. GIE 2012

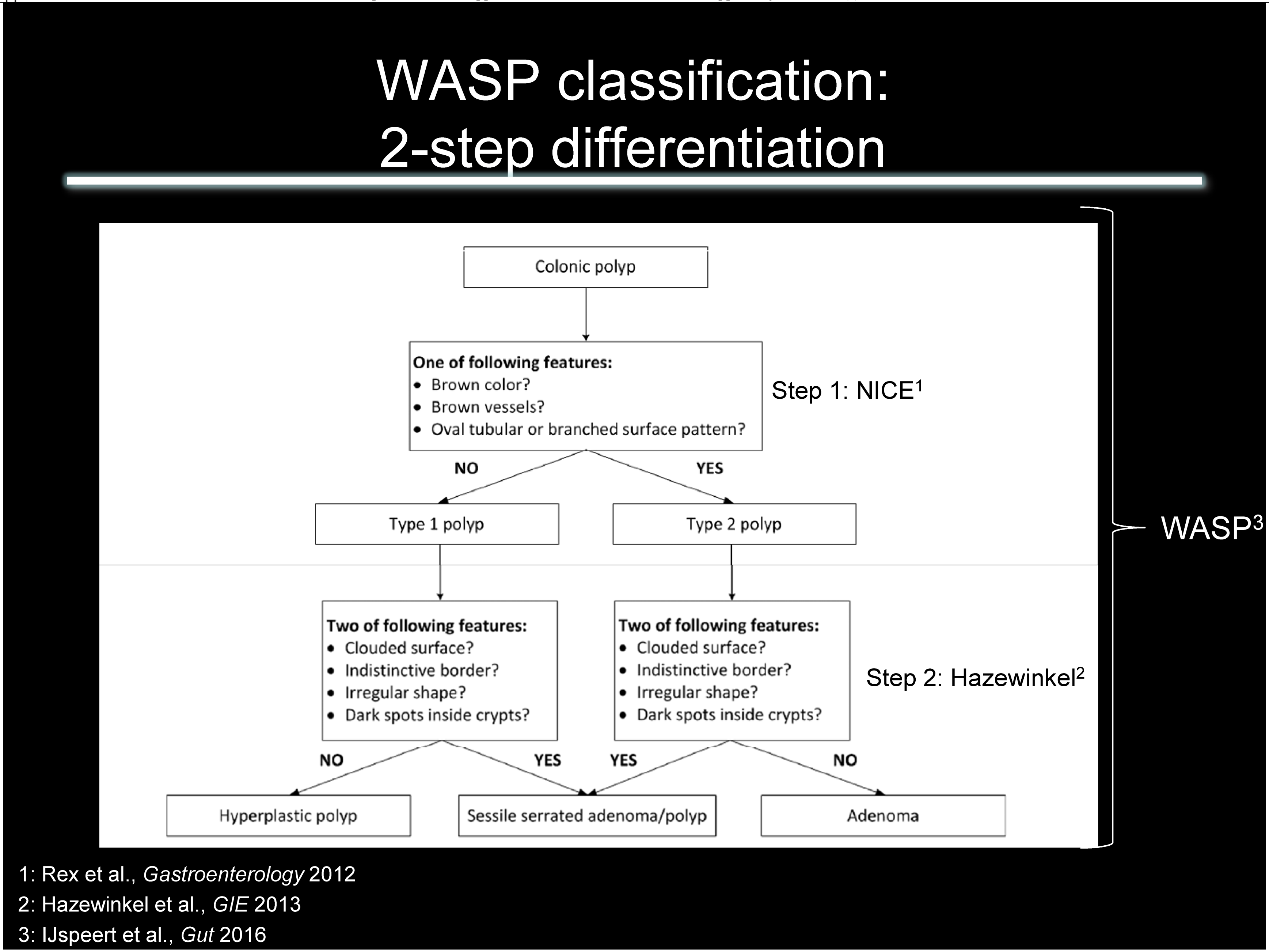
WASP classification:
2-step differentiation
Colonic polyp
One of following features:
Brown color?
Brown vessels?
Oval tubular or branched surface pattern?
Step 1: NICE1
NO
YES
Type 1 polyp
Type 2 polyp
WASP3
Two of following features:
Clouded surface?
Indistinctive border?
Irregular shape?
Dark spots inside crypts?
Two of following features:
Clouded surface?
Indistinctive border?
Irregular shape?
Dark spots inside crypts?
Step 2: Hazewinkel2
NO
YES
YES
NO
Hyperplastic polyp
Sessile serrated adenoma/polyp
Adenoma
1: Rex et al., Gastroenterology 2012
2: Hazewinkel et al., GIE 2013
3: IJspeert et al., Gut 2016

BMJ Publishing Group Limited (BMJ) disclaims all liability and responsibility arising from any reliance placed on this supplemental material which has been supplied by the author(s)

STEP 1:
NICE CLASSIFICATION

BMJ Publishing Group Limited (BMJ) disclaims all liability and responsibility arising from any reliance placed on this supplemental material which has been supplied by the author(s)

Characteristic 1: Color

Hyperplastic polyp	Adenoma
Same or lighter than background	Browner relative to background

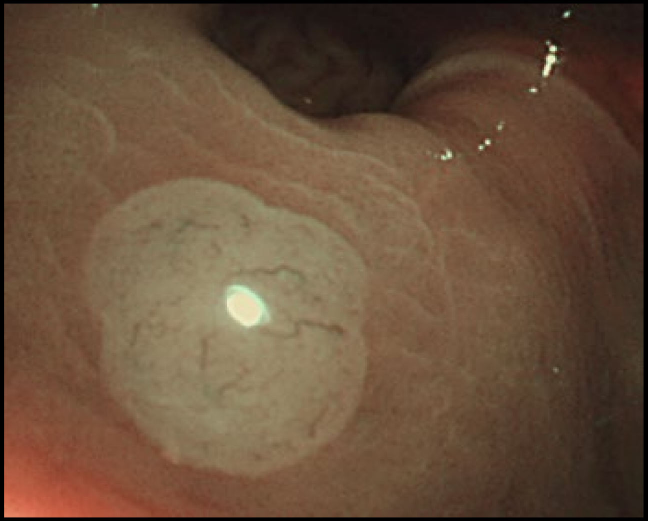

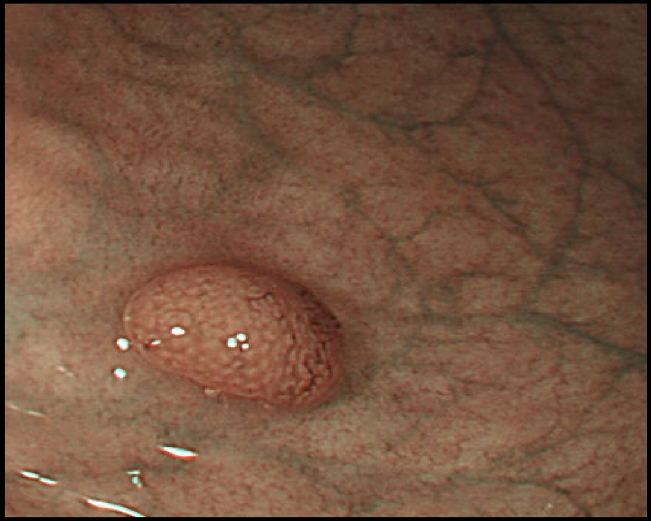

Characteristic 2: Vessels

Hyperplastic polyp	Adenoma
None, or isolated lacy vessels	Brown vessels surrounding white structures

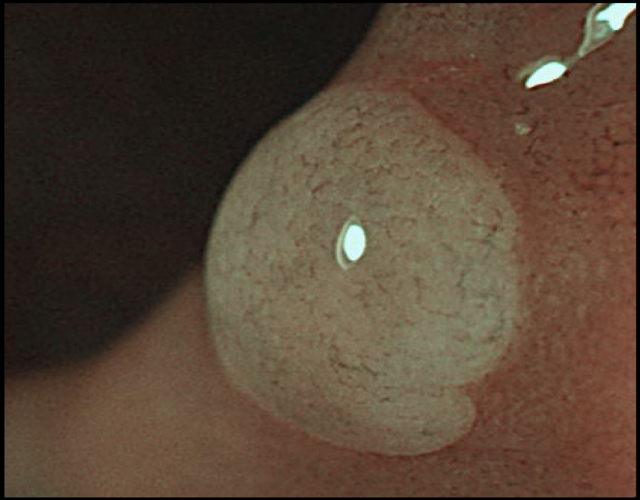

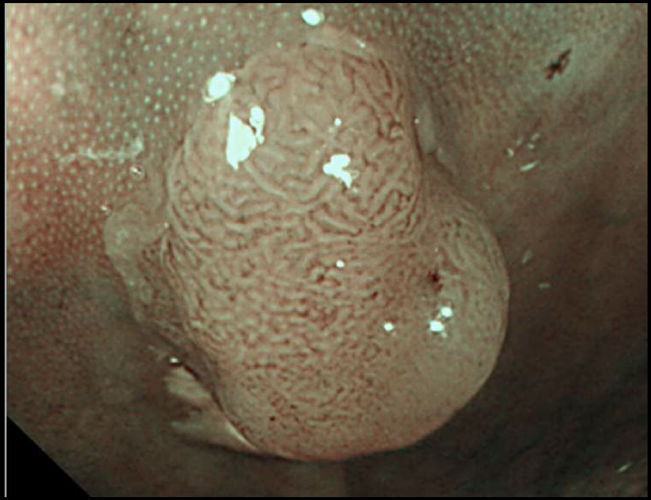

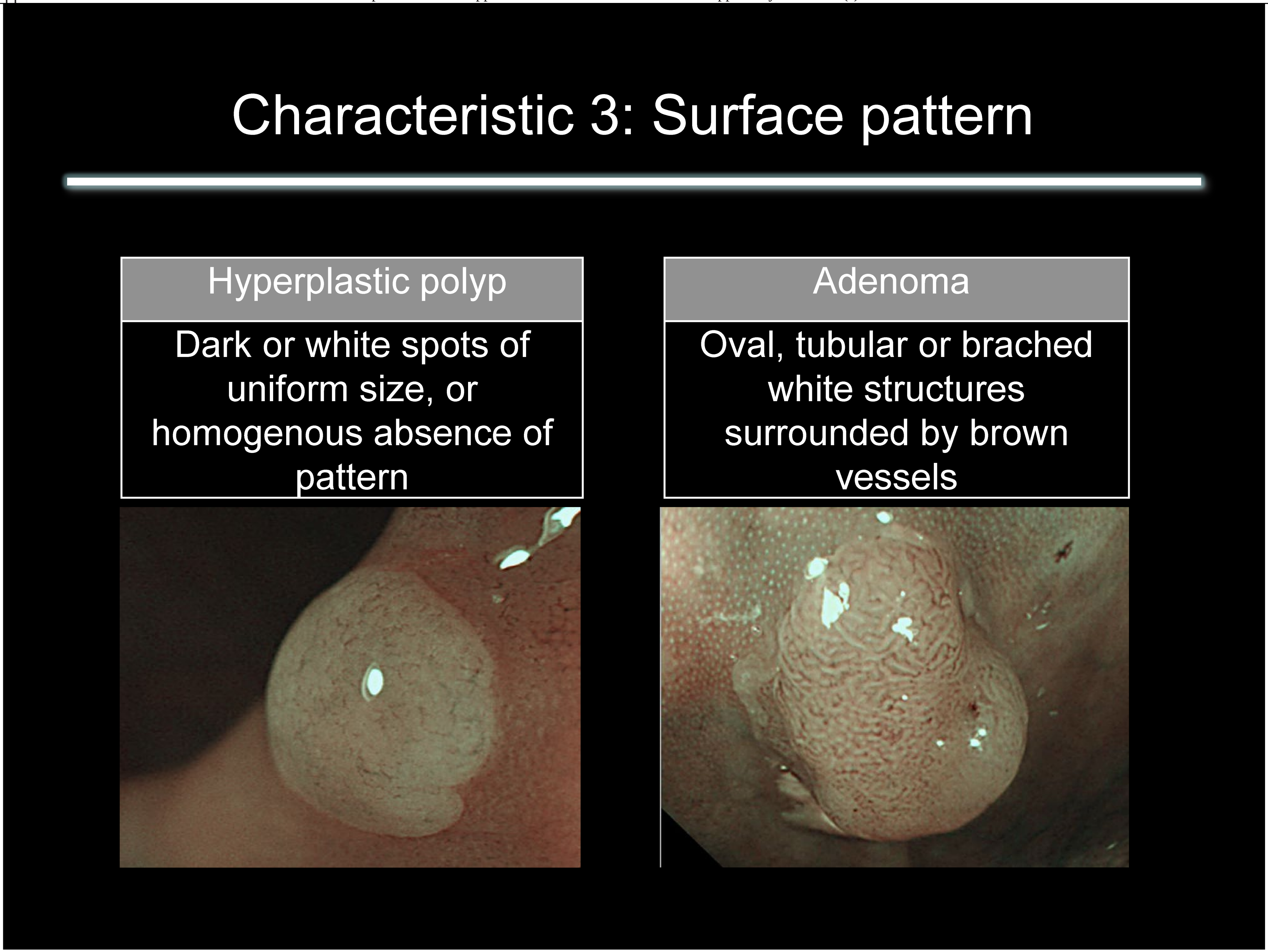
Characteristic 3: Surface pattern
Hyperplastic polyp
Dark or white spots of uniform size, or homogenous absence of pattern
Adenoma
Oval, tubular or brached white structures surrounded by brown vessels

STEP 2:
HAZEWINKEL CLASSIFICATION

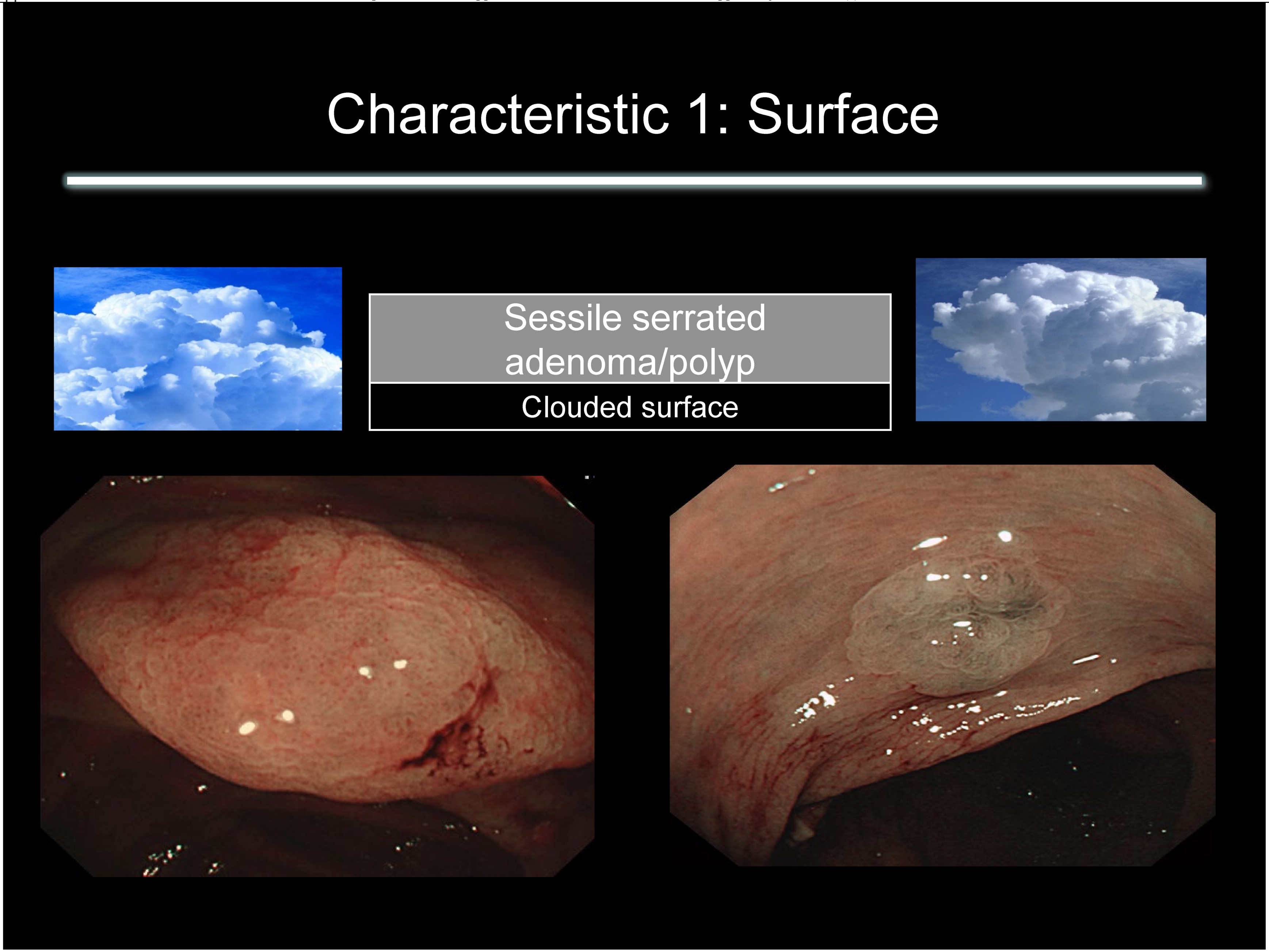
Characteristic 1: Surface
Sessile serrated adenoma/polyp
Clouded surface

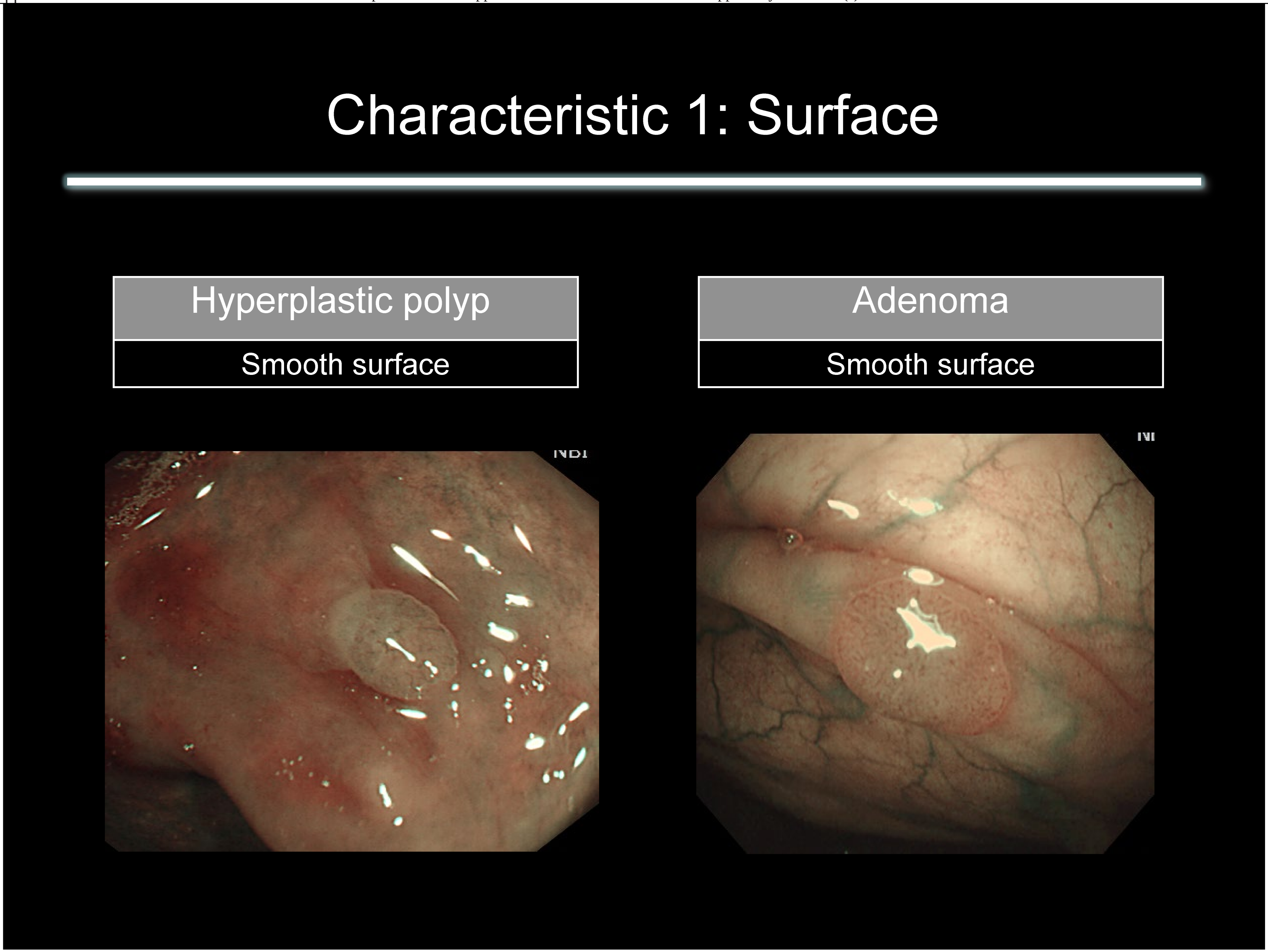
Characteristic 1: Surface
Hyperplastic polyp
Smooth surface
Adenoma
Smooth surface

Characteristic 2: Border

Sessile serrated adenoma/polyp
Indistinct (vague) border

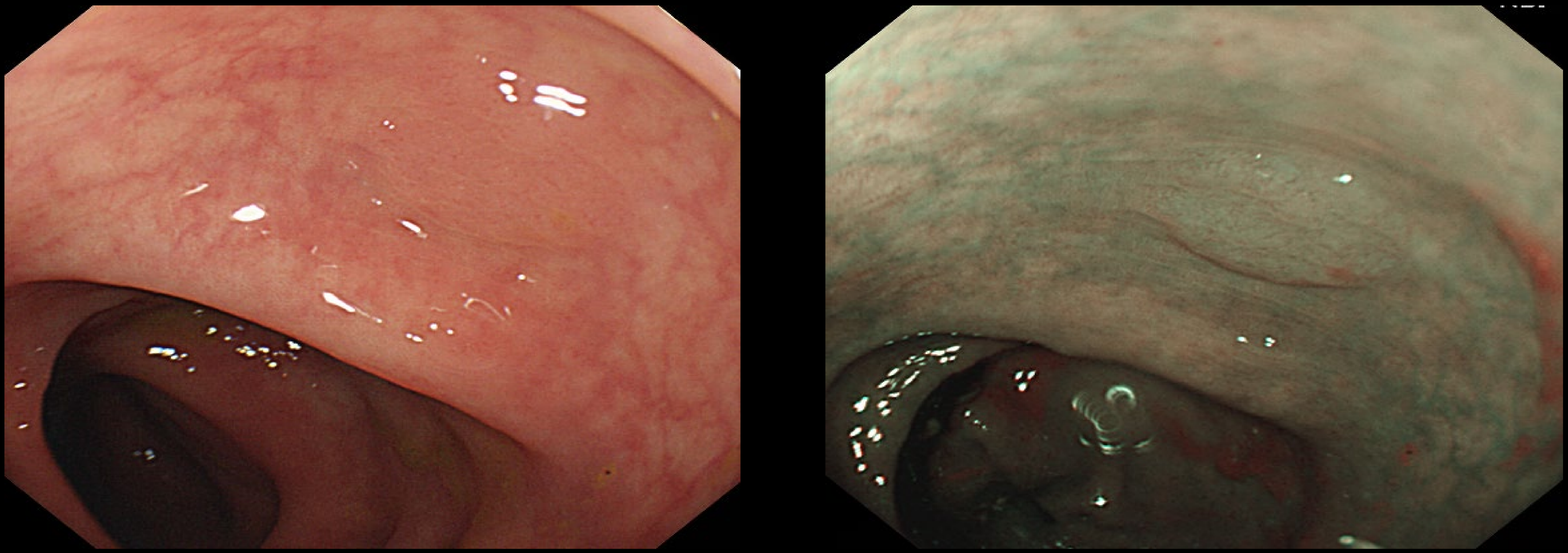

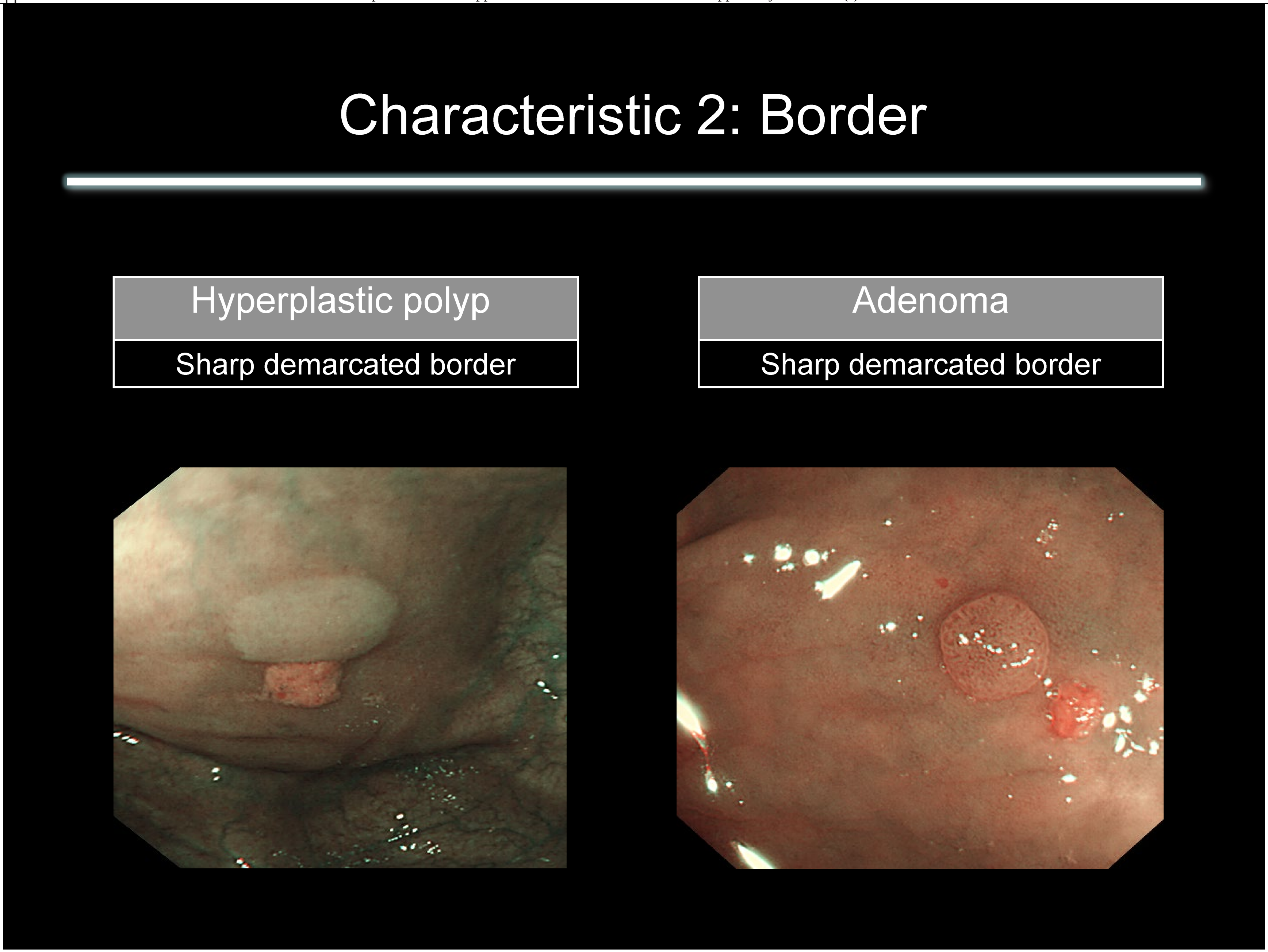
Characteristic 2: Border
Hyperplastic polyp
Sharp demarcated border
Adenoma
Sharp demarcated border

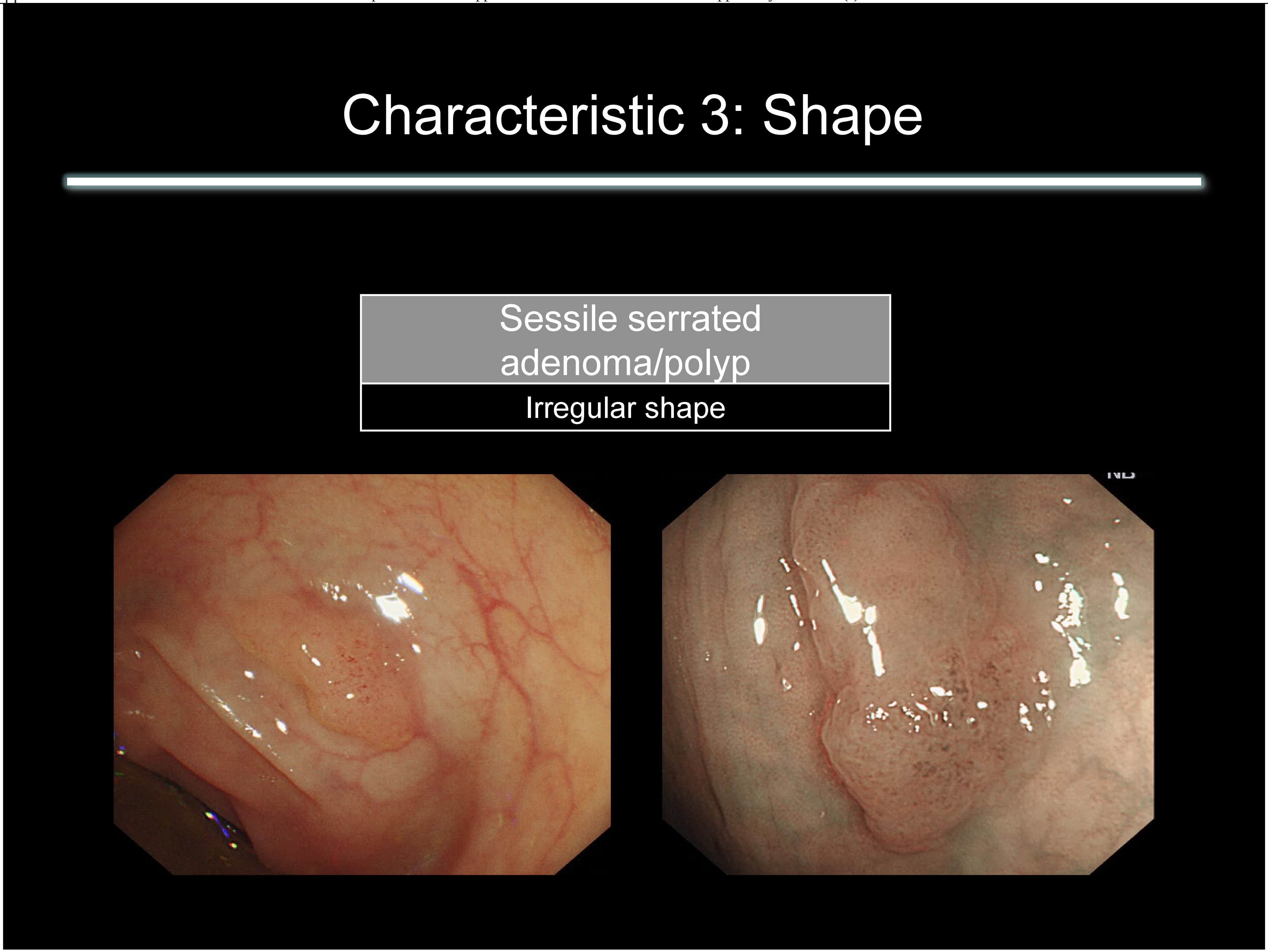
Characteristic 3: Shape
Sessile serrated adenoma/polyp
Irregular shape

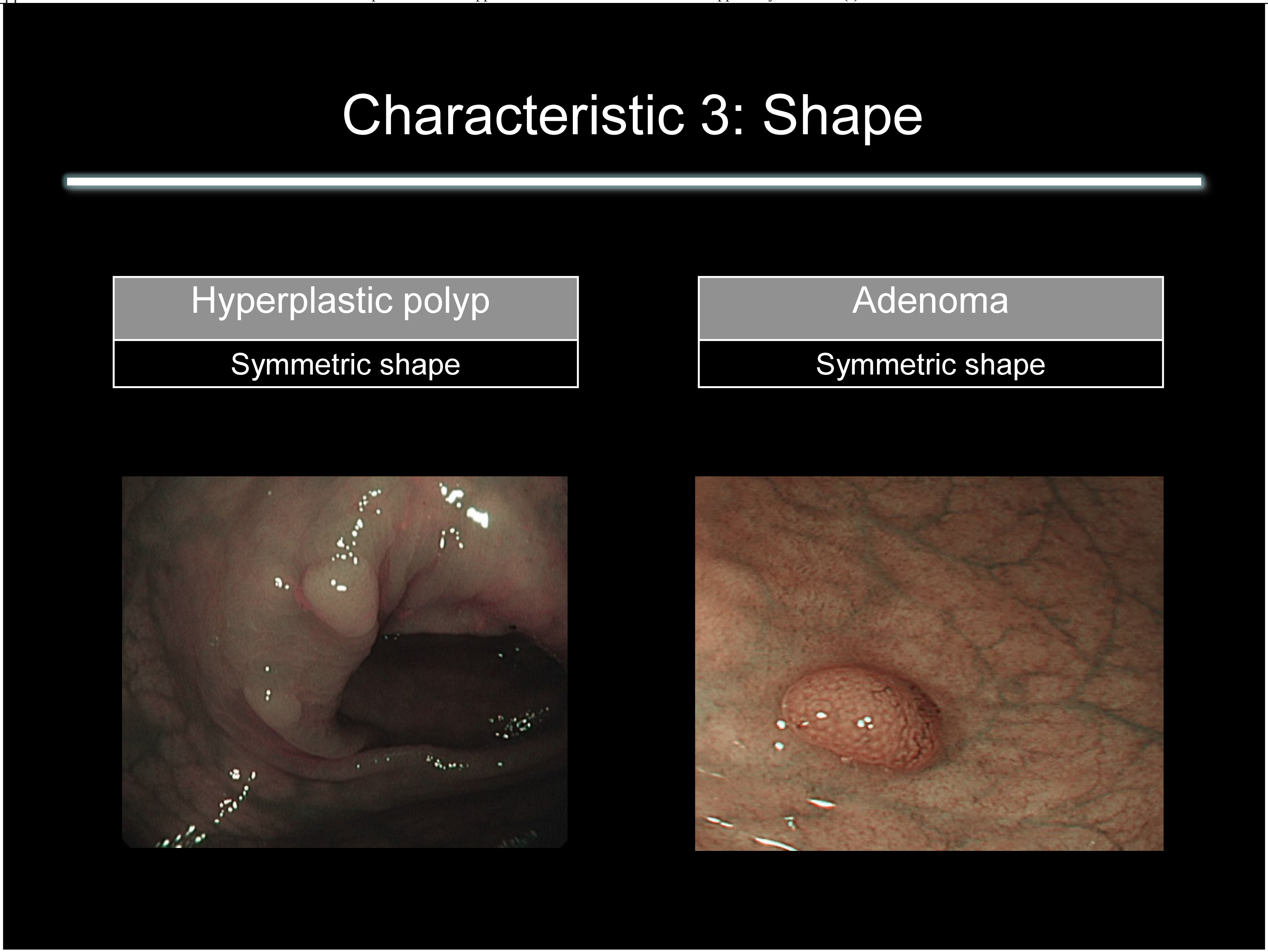
Characteristic 3: Shape
Hyperplastic polyp
Symmetric shape
Adenoma
Symmetric shape

Characteristic 4: Crypts

Sessile serrated adenoma/polyp
Dark spots inside the crypts

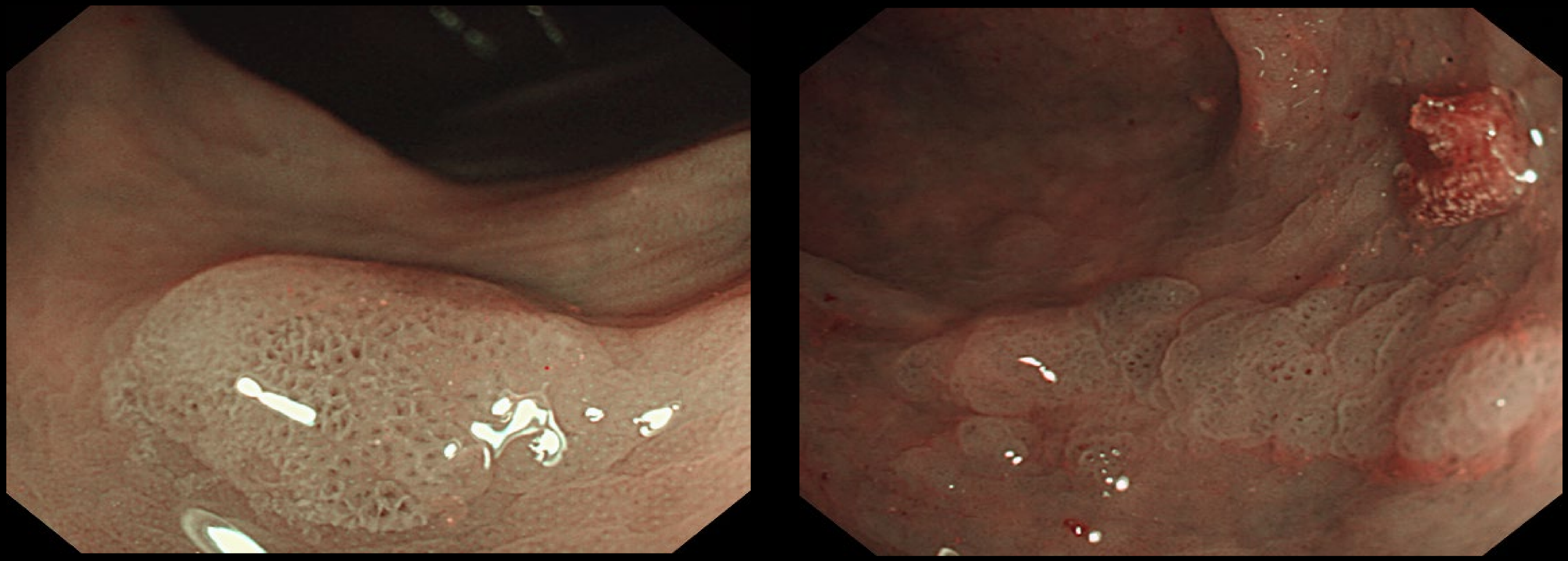

BMJ Publishing Group Limited (BMJ) disclaims all liability and responsibility arising from any reliance placed on this supplemental material which has been supplied by the author(s)

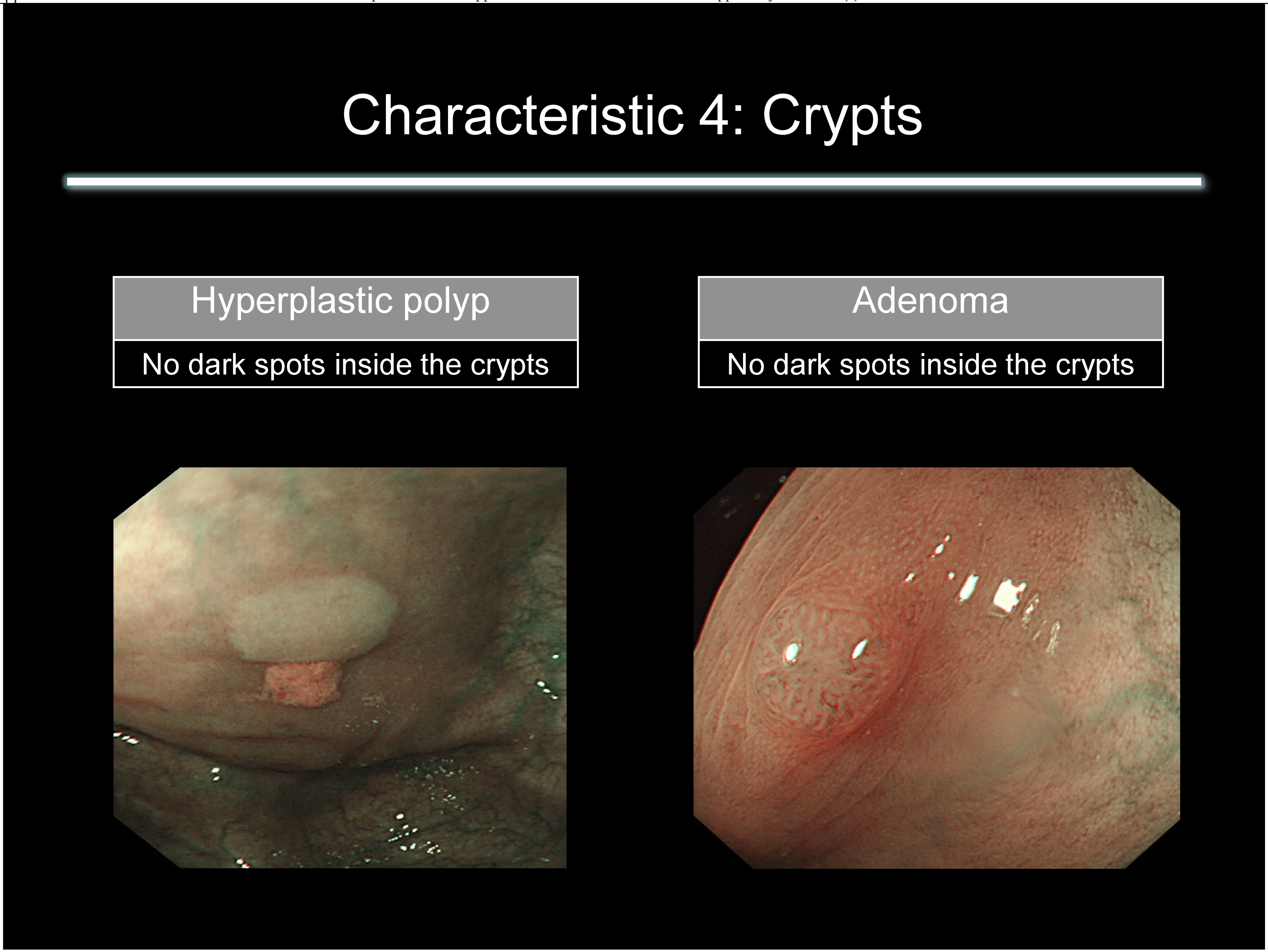

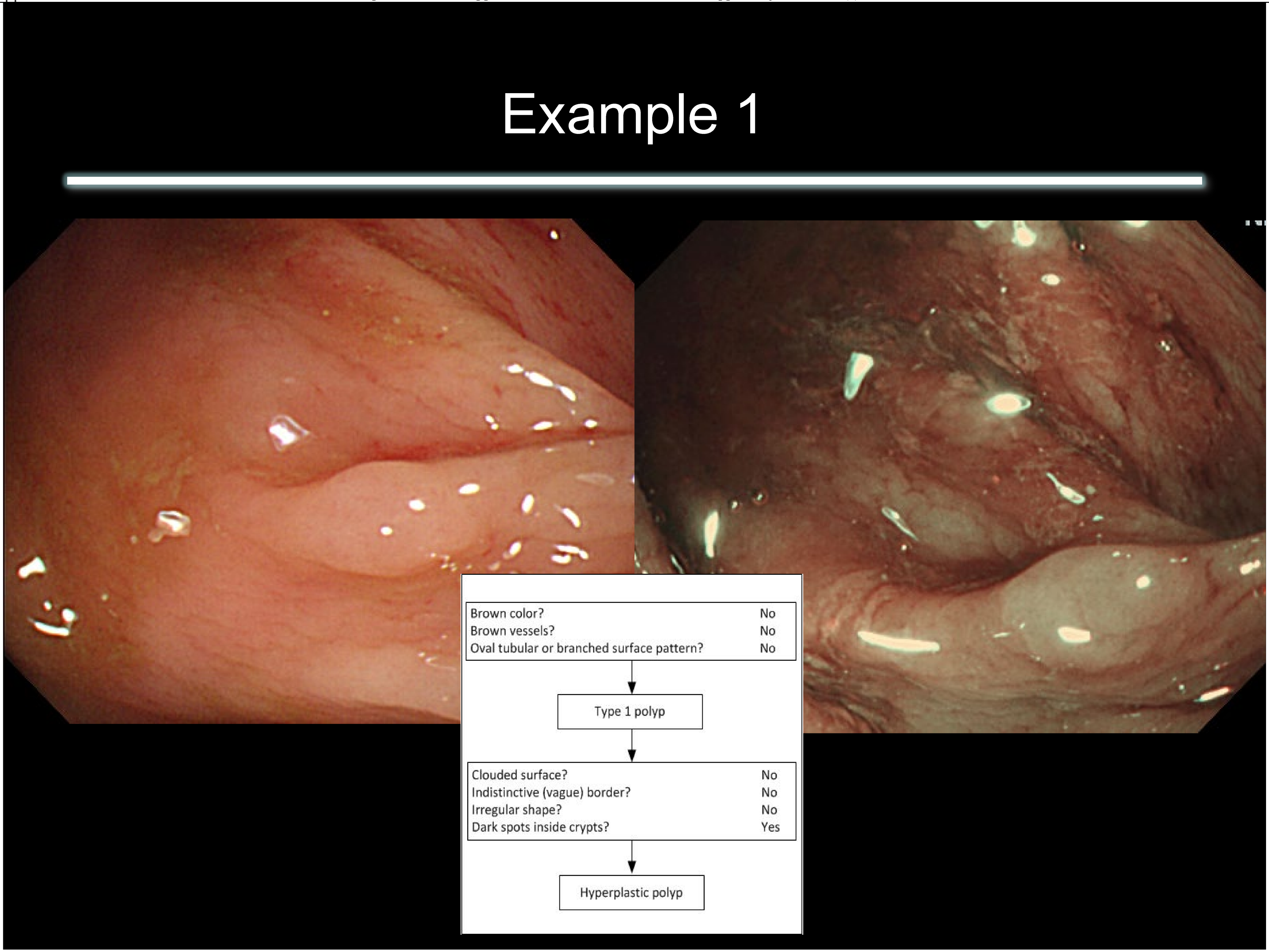
Example 1
Brown color? No
Brown vessels? No
Oval tubular or branched surface pattern? No
Type 1 polyp
Clouded surface? No
Indistinctive (vague) border? No
Irregular shape? No
Dark spots inside crypts? Yes
Hyperplastic polyp

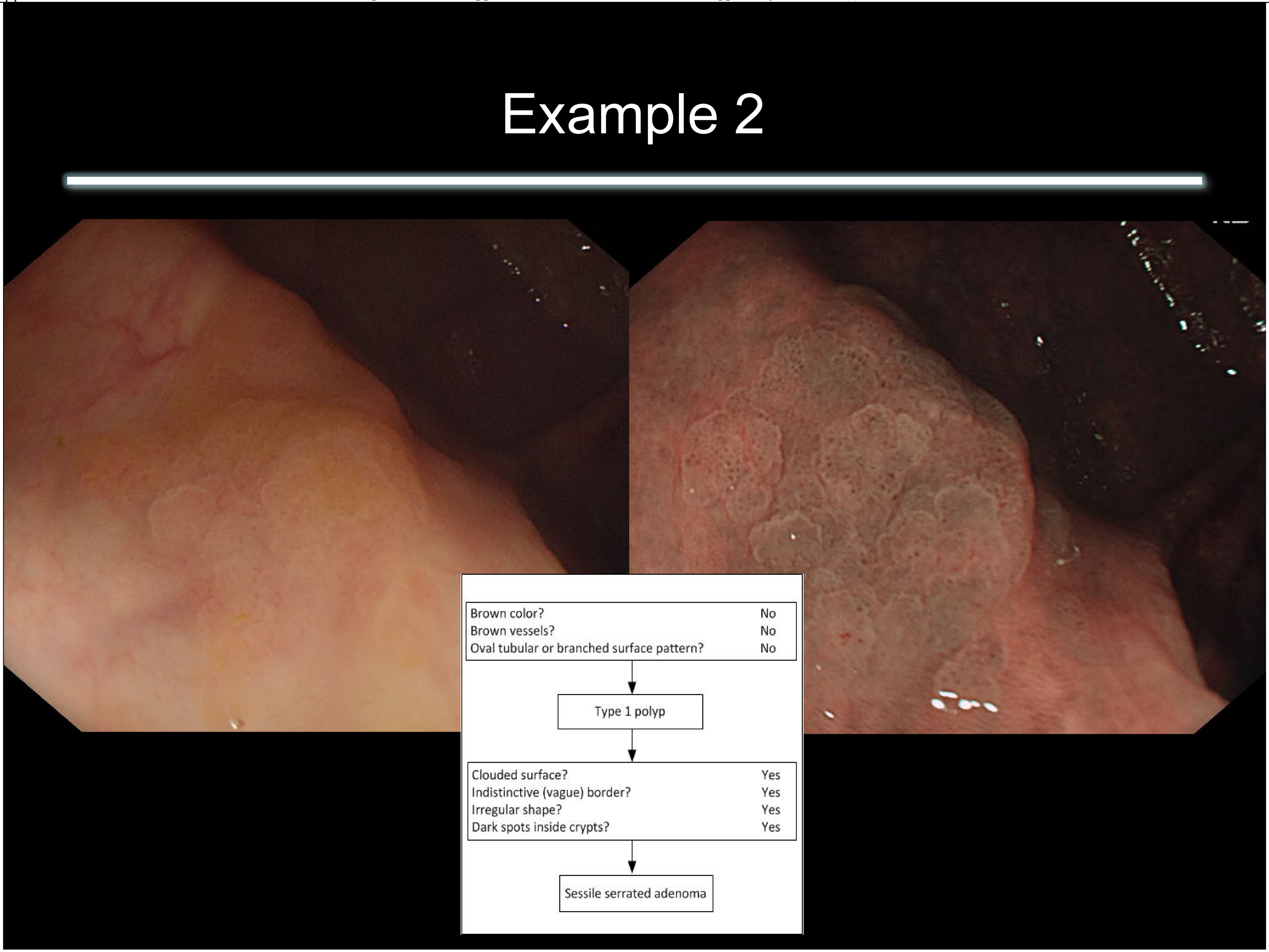
Example 2
Brown color? No
Brown vessels? No
Oval tubular or branched surface pattern? No
Type 1 polyp
Clouded surface? Yes
Indistinctive (vague) border? Yes
Irregular shape? Yes
Dark spots inside crypts? Yes
Sessile serrated adenoma

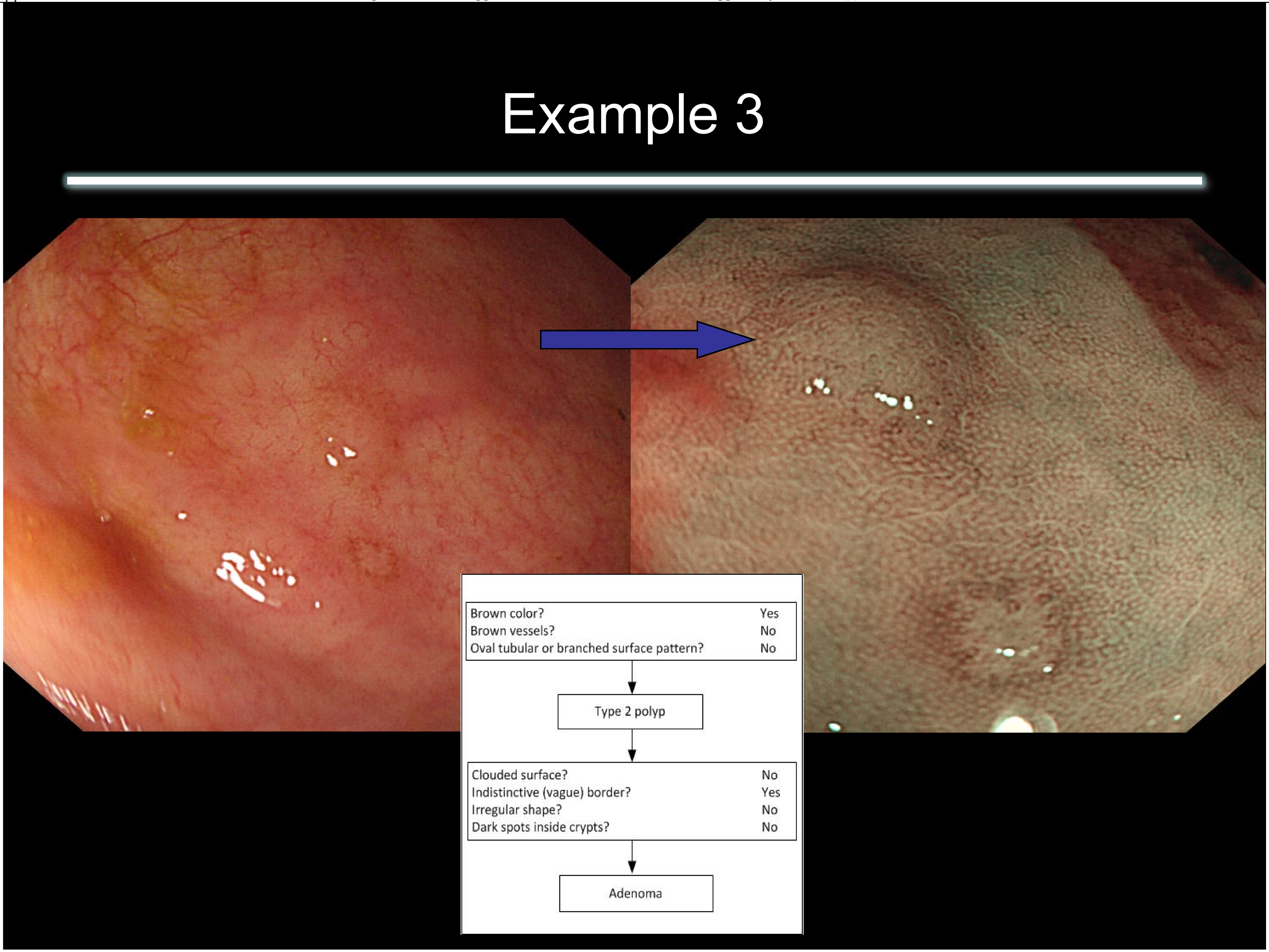
Example 3
Brown color? Yes
Brown vessels? No
Oval tubular or branched surface pattern? No
Type 2 polyp
Clouded surface? No
Indistinctive (vague) border? Yes
Irregular shape? No
Dark spots inside crypts? No
Adenoma

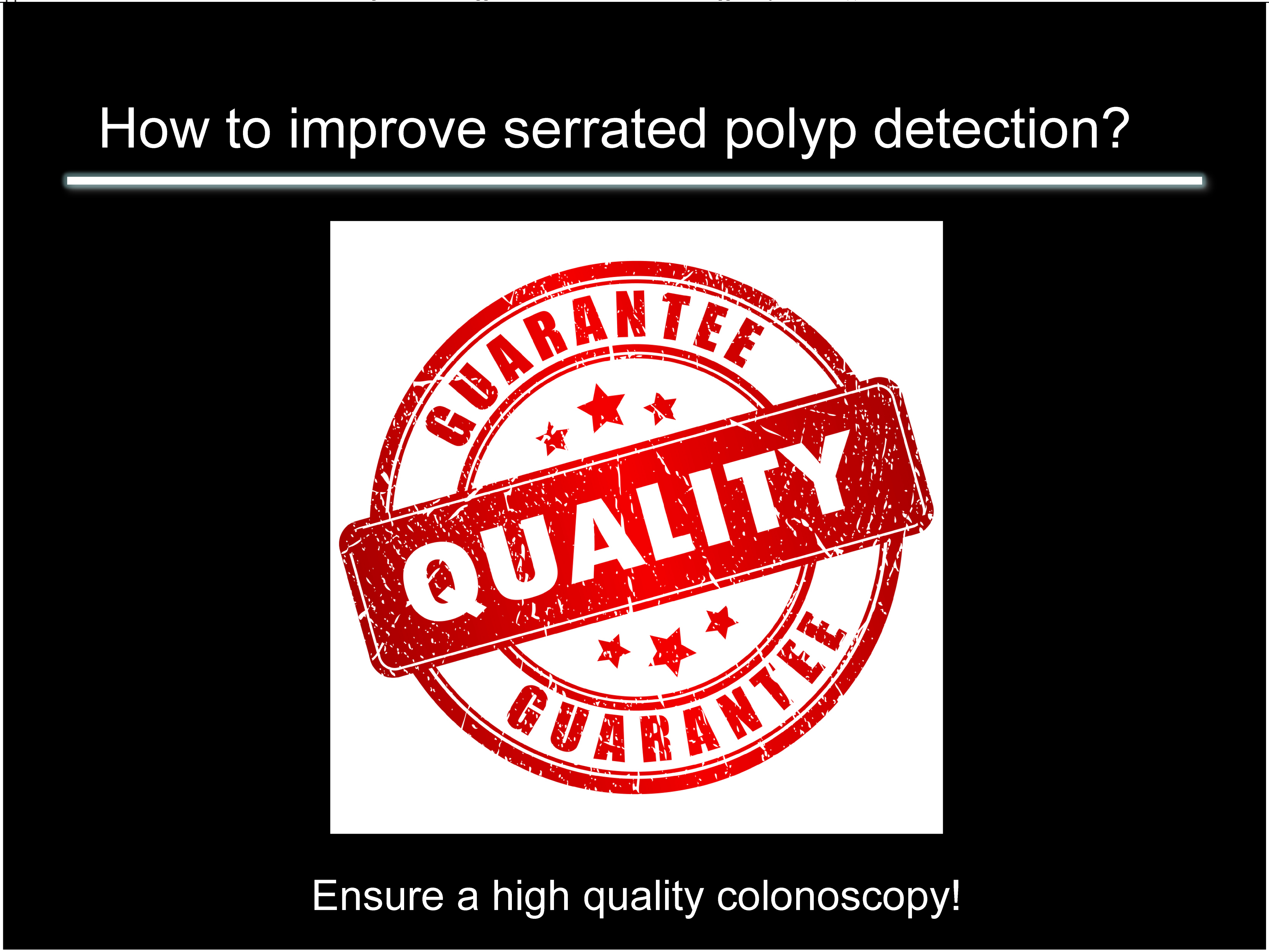
How to improve serrated polyp detection?
GUARANTEE
QUALITY
GUARANTEE
Ensure a high quality colonoscopy!

BMJ Publishing Group Limited (BMJ) disclaims all liability and responsibility arising from any reliance placed on this supplemental material which has been supplied by the author(s)

Quality of bowel preparation

- Mucus-cap of SP attracts stool causing difficulties in detection
- In clean colon the mucus-cap can help to identify SPs

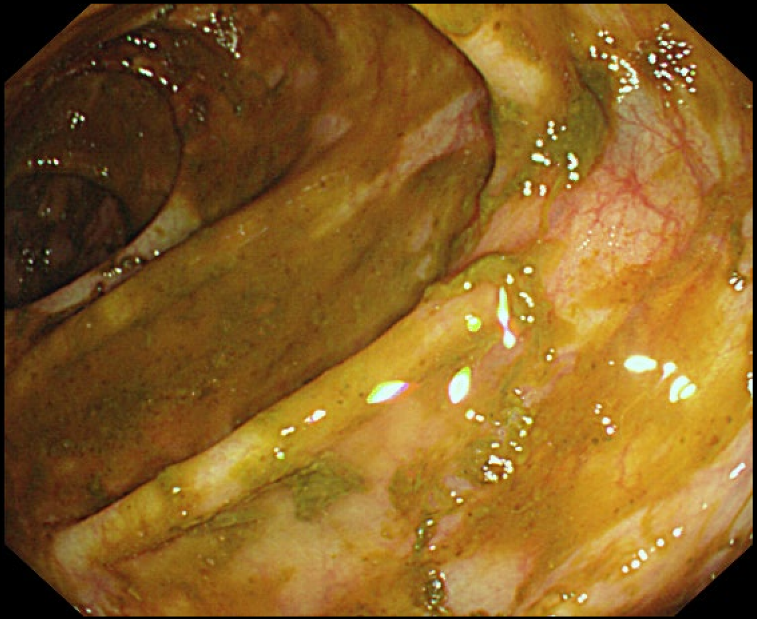

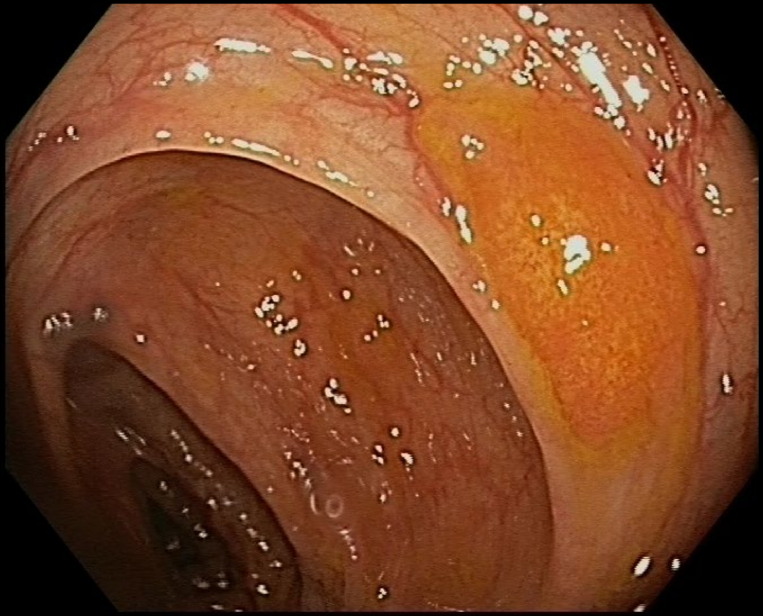

Know what to look for!

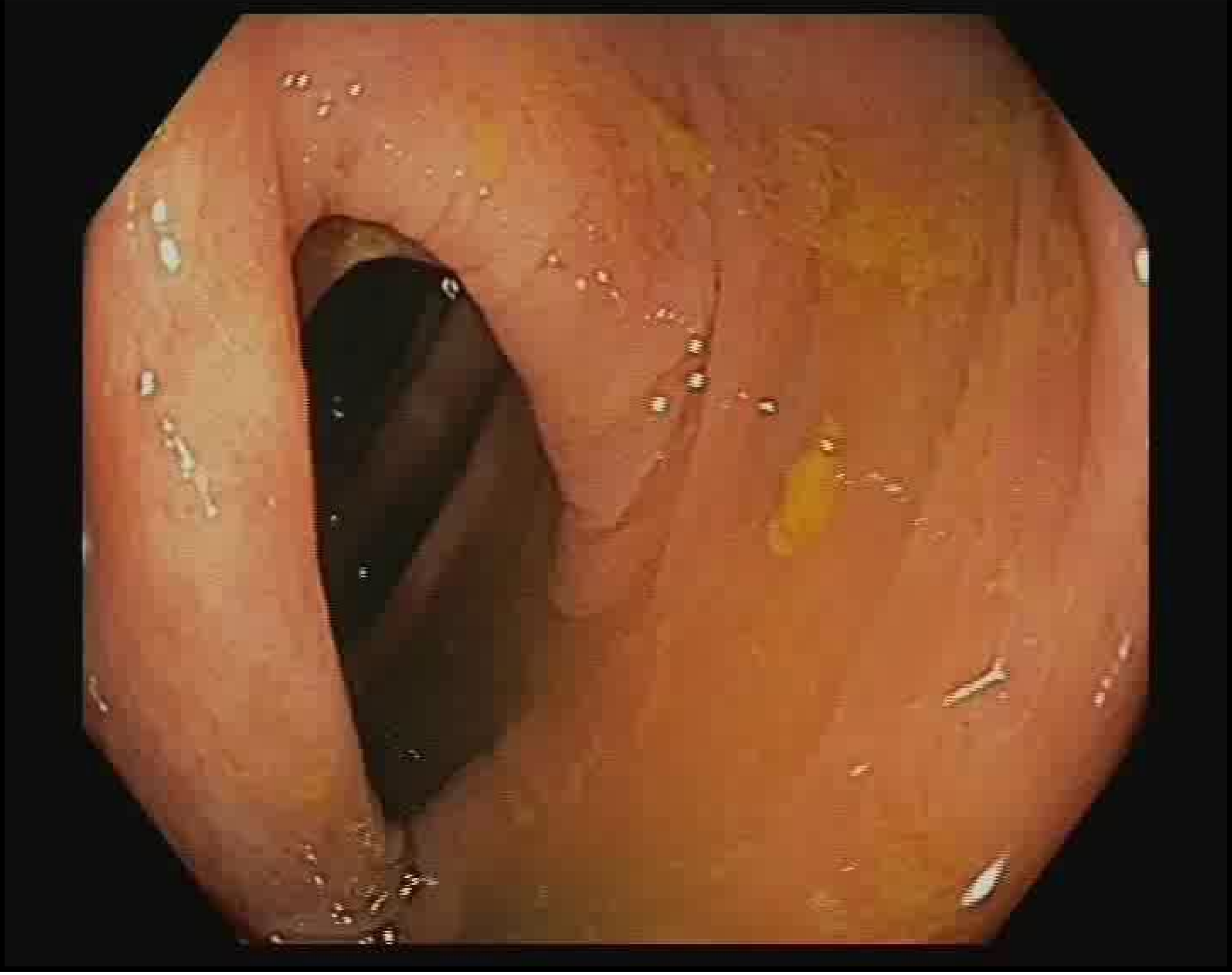

Cecal intubation

- SSA/P are more often located in the proximal colon
- Incomplete colonoscopy will result in substantial amount of missed SPs and subsequent interval CRC

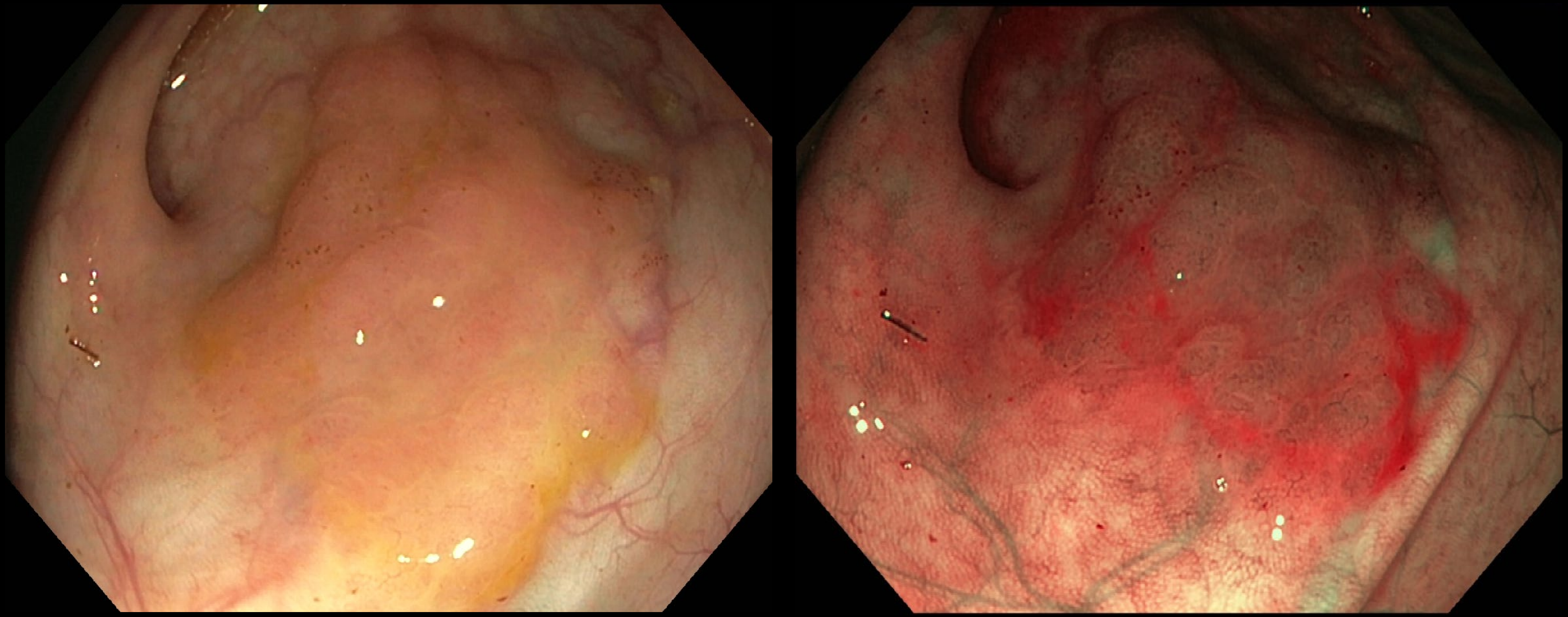

NHS BCSP Endosc Qual Assurance group 2011

Adequate withdrawal time

- Longer withdrawal time results in higher detection of SP

ORIGINAL ARTICLE: Clinical Endoscopy

Differences in proximal serrated polyp detection among endoscopists are associated with variability in withdrawal time (CME)

Thomas R. de Wijkerslooth, MD,[1] Esther M. Stoop, MD,[2] Patrick M. Bossuyt, PhD,[3] Kristien M.A.J. Tytgat, MD, PhD,[1] Jan Dees, MD,[2] Elisabeth M.H. Mathus-Vliegen, MD, PhD,[1] Ernst J. Kuipers, MD, PhD,[2] Paul Fockens, MD, PhD,[1] Monique E. van Leerdam, MD, PhD,[2] Evelien Dekker, MD, PhD[1]

Rotterdam, Amsterdam, The Netherlands

de Wijkerslooth et al. GIE 2013

Role of advanced imaging in SP detection

- Narrow Band Imaging (NBI) has no proven additional value in SP detection in SPS patients
- Chromoendoscopy (CE) probably usefull but time consuming
- Advanced imaging usefull to detect SPs in case of uncertainty!

Hazewinkel et al. GIE 2014
Fu et al. Int J Clin Exp Pathol. 2014

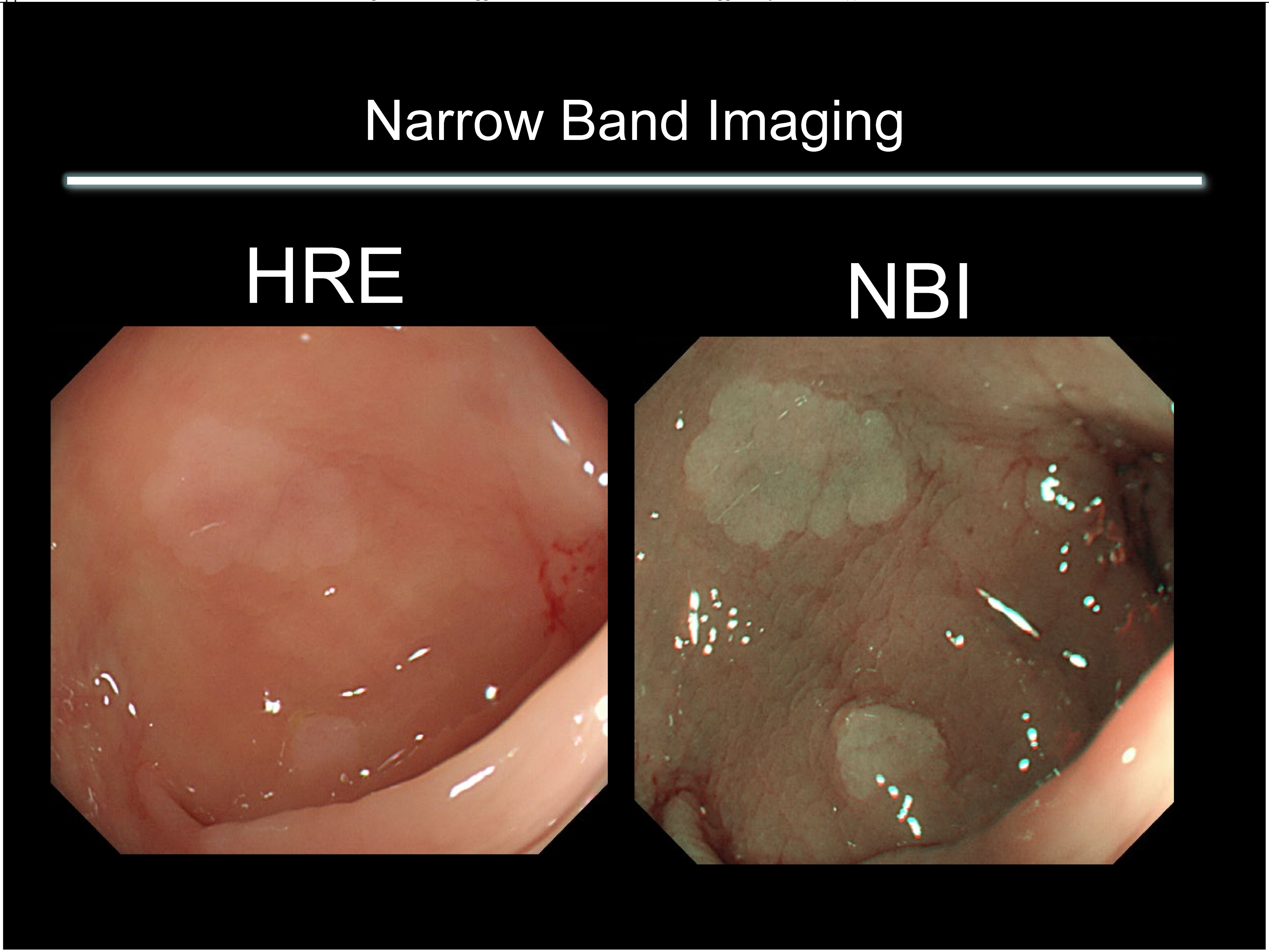
Narrow Band Imaging
HRE
NBI

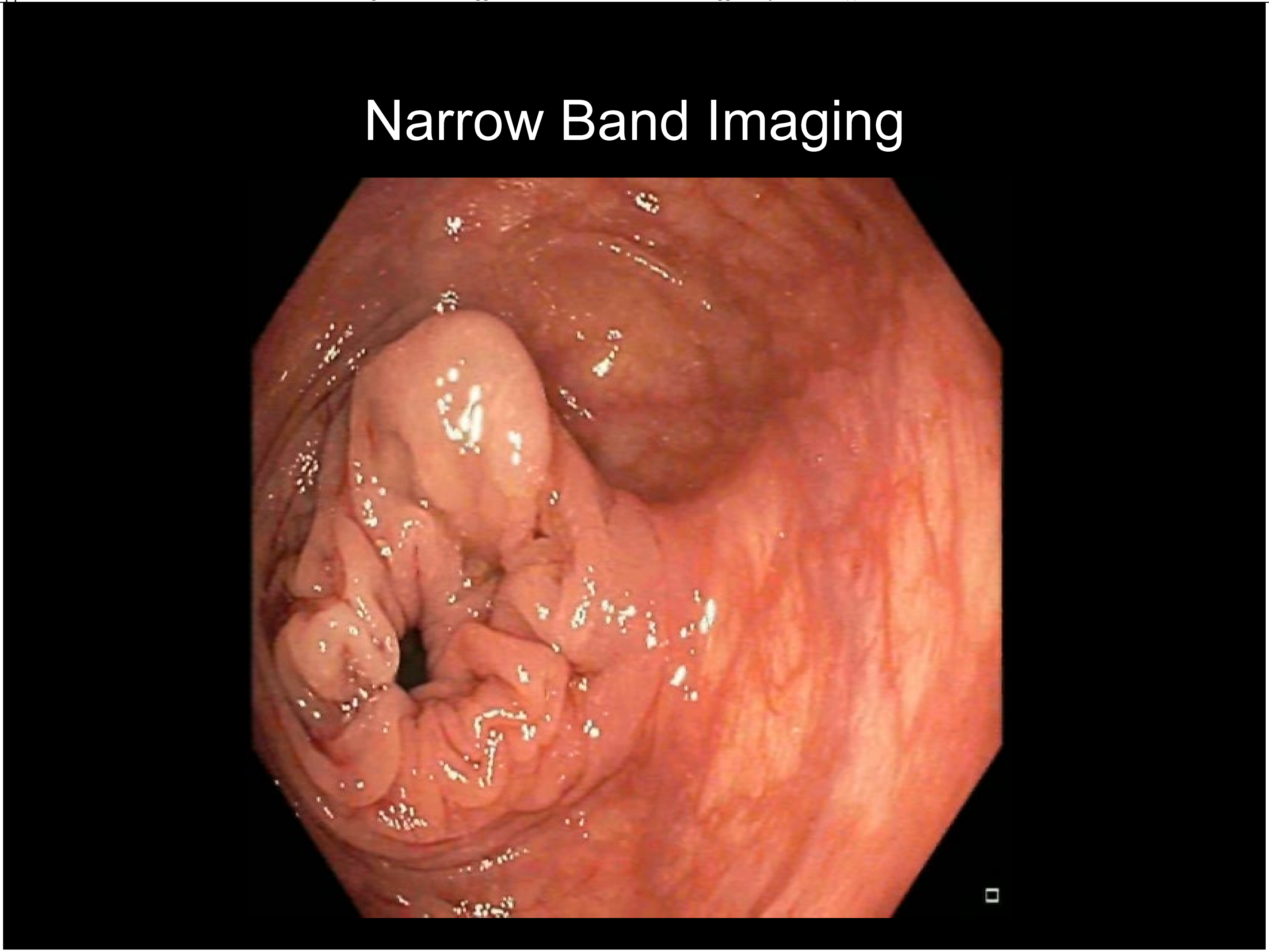
Narrow Band Imaging

Conclusion

- SPs are easily missed during colonoscopy
- Important reason for right-sided interval CRC
- SP detection can be improved by:
  - Awareness of malignant potential SPs
  - Knowledge of subtle SP features
  - High quality colonoscopy
  - Targeted use of advanced imaging techniques
