# Supplementary material for: Substantial and sustained improvement of serrated polyp detection after a simple educational intervention: results from a prospective controlled trial
Source: Gut. 2020 Mar 5;69(12):2150–8. doi: 10.1136/gutjnl-2019-319804 (PMC7677479; doi:10.1136/gutjnl-2019-319804)
Supplement: Supplementary data [file gutjnl-2019-319804supp002.pdf]

Supplementary table 1 – Sensitivity analysis including endoscopists present during only one of the two training sessions.

	Untrained endoscopists (control group)	Trained endoscopists, attended both training sessions	Trained endoscopists, attended only one training session
**Number of endoscopists**	100	17	11
**Number of colonoscopies**			
Baseline	6,069	928	349
Post-training *	44,967	10,377	3464
**Baseline PSPDR**	9.3% (8.6-10%)	9.3% (7.4-11%)	10.6% (7.6-14%)
**Post-training PSPDR***	10.2% (10.0-10.5%)	13.8% (13.2-14.5)	12.8% (12-14%)
**OR for detection of ≥1 PSP**	1.00 (reference)	1.49 (1.08-2.06)	1.37 (0.91-2.06)

*This represents colonoscopies performed in 2015 onwards (control endoscopists), or colonoscopies performed after first attended training session.
