# Supplementary figures and images for: Substantial and sustained improvement of serrated polyp detection after a simple educational intervention: results from a prospective controlled trial
Source: Gut. 2020 Mar 5;69(12):2150–8. doi: 10.1136/gutjnl-2019-319804 (PMC7677479; doi:10.1136/gutjnl-2019-319804)

Schematic overview of invitations based on to birth year

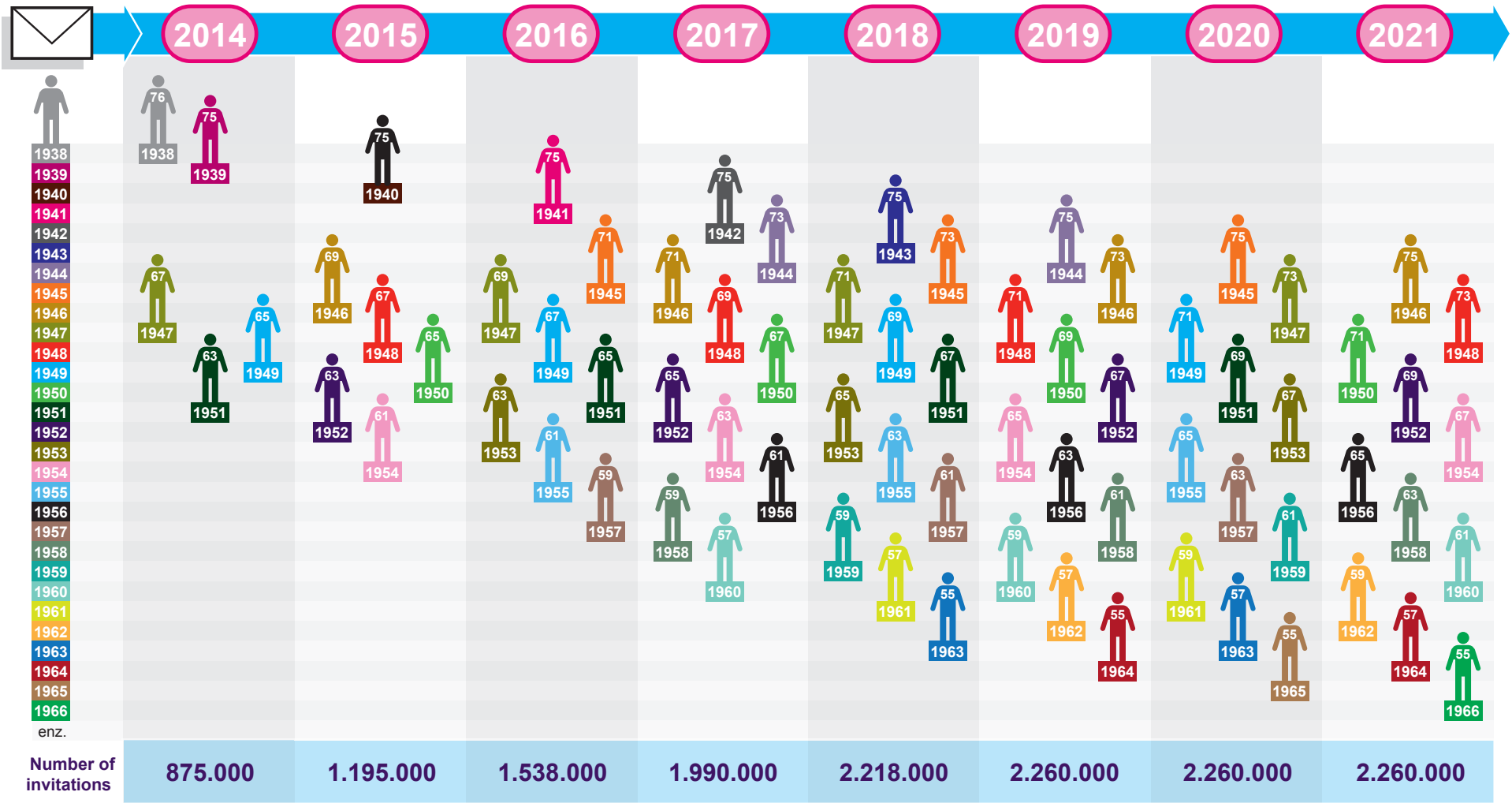

Supplement: Supplementary data [file gutjnl-2019-319804supp003.pdf]
